# AtSNU13 modulates pre-mRNA splicing of *RBOHD* and *ALD1* to regulate plant immunity

**DOI:** 10.1186/s12915-024-01951-9

**Published:** 2024-07-10

**Authors:** Yanke Jiang, Yingzhe Yue, Chongchong Lu, Muhammad Zunair Latif, Haifeng Liu, Zhaoxu Wang, Ziyi Yin, Yang Li, Xinhua Ding

**Affiliations:** 1 State Key Laboratory of Crop Biology, Shandong Provincial Key Laboratory for Biology of Vegetable Diseases and Insect Pests, College of Plant Protection, Shandong Agricultural University, Tai an, Shandong 271018 China; 2 State Key Laboratory of Crop Biology, College of Agronomy, Shandong Agricultural University, Taian, Shandong 271018 China

**Keywords:** Pattern-triggered immunity, Effector-triggered immunity, RNA splicing, Post-transcriptional regulation, Spliceosome component, Plant immunity

## Abstract

Pre-mRNA splicing is a significant step for post-transcriptional modifications and functions in a wide range of physiological processes in plants. Human NHP2L binds to U4 snRNA during spliceosome assembly; it is involved in RNA splicing and mediates the development of human tumors. However, no ortholog has yet been identified in plants. Therefore, we report At4g12600 encoding the ortholog *NHP2L* protein, and *AtSNU13* associates with the component of the spliceosome complex; the *atsnu13* mutant showed compromised resistance in disease resistance, indicating that *AtSNU13* is a positive regulator of plant immunity. Compared to wild-type plants, the *atsnu13* mutation resulted in altered splicing patterns for defense-related genes and decreased expression of defense-related genes, such as *RBOHD* and *ALD1*. Further investigation shows that AtSNU13 promotes the interaction between U4/U6.U5 tri-snRNP-specific 27 K and the motif in target mRNAs to regulate the RNA splicing. Our study highlights the role of *AtSNU13* in regulating plant immunity by affecting the pre-mRNA splicing of defense-related genes.

**Supplementary Information:**

The online version contains supplementary material available at 10.1186/s12915-024-01951-9.

## Background

Plants constantly interact with a wide range of microorganisms during their life, which may affect their fecundity, yield, and lifespan. Two types of immunity have evolved to avoid these potential attacks and resist the pathogen’s invasion in the host. The first line of defense is activated by the pathogen-associated molecular patterns (PAMPs or MAMPs), and PAMP-triggered immunity (PTI), which is a basal defense in plants, is recognized by pattern recognition receptors (PRRs) [1, 2]. However, to overcome PTI, pathogens deliver effectors into host plant cells. Similarly, plants have developed resistance (R) proteins to recognize avirulence (AVR) effectors from pathogens to activate intracellular immunity [3]. The R protein, which typically contains a central nucleotide-binding site (NBS) and a C-terminal leucine-rich repeat (LRR) region, confers recognition specificity, resulting in the second line of defense, known as effector-triggered immunity (ETI) [4]. The production of reactive oxygen species is a marker of early PTI response in plants. The receptors of PTI and the key genes for the production of reactive oxygen species also play important roles in the ETI signaling pathway. Studies have shown that plant immune receptors PRRs and NLRs are necessary for the production of reactive oxygen species in ETI [5, 6]. The defensive signaling pathways of ETI and PTI have long-term similarities. The interaction of cell-surface and intracellular receptors in plant defense enables plants to construct more complex defense mechanisms.

Pre-mRNA splicing is an important step for gene expression, gene regulation, and protein biosynthesis during the plant immune system activation, generating more than one mature mRNA from a single gene [7–9]. The frequency of splicing varies among species, i.e., intron retention is the abundant type of differential alternative splicing in *Arabidopsis thaliana*, maize, and other plants, while exon skipping occurs a little less frequently [10]. Splicing, on the other hand, can result in the production of truncated proteins by destroying the open reading frame (ORF) of genes [11], as well as triggering nonsense-mediated mRNA decay (NMD), both of which lead to the downregulation of gene expression [12].

In *Arabidopsis* [13] and rice [14], more than 60% and 48% of mature mRNAs are generated by RNA splicing, respectively [15], and more new splice sites appear under stress conditions [16, 17]. Alternative splicing is significantly altered during abiotic and biotic stress, which is a novel regulation mechanism to rapidly reprogram gene expression [18–21]. The splicing process is carried out by a large ribonucleoprotein complex known as the spliceosome. A complete ribonucleoprotein complex consists of five highly conserved small nuclear ribonucleoproteins (snRNPs): U1, U2, U4, U5, and U6, as well as over 300 other proteins [22]. These proteins include serine/arginine (SR)-rich proteins, heterogeneous nuclear ribonucleoproteins (hnRNPs), and RNA-binding proteins (RBPs) [23]. In addition to snRNPs, many non-snRNPs, such as splicing factors SF1/mBBP and U2AF, are involved in the recognition of the 3′ splice site at the early stage of spliceosome assembly [24]. Non-snRNPs regulating pre-mRNA splicing and alternative splicing is a significant step in plant development and response to environmental stimuli [25–28]. Mutations of splicing factors, spliceosome components, and spliceosome assembly factors usually contribute to an increased frequency of RNA splicing [29, 30].

RNA splicing regulates different aspects of plant immunity, like the modulation of key immunity receptors [31, 32], *R* genes [33], and key genes of hormone signaling pathways [34–36]. In addition, the role of the modulation of splicing factors in plant immunity is also notable [37]. Plant cell-surface receptor pre-mRNA splicing generates different transcripts that can induce pathogen infection by influencing downstream signal transduction. For example, flg22 is a conserved bacterial flagellin domain that can be detected by plant Flagellin-sensing 2 (FLS2), a classic PRR protein [38, 39]. Alternative splicing of the first exon of *FLS2* regulates the accumulation of transcript through a mechanism called intron-mediated enhancement (IME), and the transformed transcript can encode the repressor in the FLS2 pathway and affect the production of reactive oxygen species mediated by FLS2 [40].

The yeast protein Snu13p and its human ortholog were first isolated from the purified U4/U6.U5 tri-snRNP, a complex particle of the spliceosome, which was found to be associated with the 5′-terminal end stem-loop of U4 snRNA [41, 42]. In humans, Snu13 appears to be a highly conserved nuclear protein that is a component of the [U4/U6.U5] tri-snRNP. Moreover, it was reported that CmSnu13 (*Cyanidioschyzon merolae*) is a key component of spliceosome and Snu13 functions in the splicing pathway [43]. In humans, *Snu13/NHP2L* acts as a core protein associated with the function of snoRNAs and mediates the development of human tumors [44, 45]. However, the plant homolog of Snu13 has yet to be identified. On databases such as TAIR and KEGG, the AtSNU13 gene was described and annotated as a spliceosome component and part of the U4/U6.U5 tri-snRNP complex in *A. thaliana*, and AtSnu13 (synonym 15.5 K) and other proteins were reported to interact with spliceosome components to form an active snoRNP complex [46].

In this study, we characterized an ortholog gene of the yeast *Snu13p* in *Arabidopsis*, called *AtSNU13*. We observed that the *atsnu13* homozygous mutants reduced the resistance to *Pst* DC3000 (*Pseudomonas pv.* tomato DC3000), *Pst* DC3000 (avr*Rpt2*) (strains carrying RPT2 effectors, which can active RESISTANCE P. SYRINGAE 2 (RPS2)-dependent ETI in wild-type plants), and D36E (all 36 effector genes and coronatine biosynthesis genes are deleted and is thus expected to activate only PTI). Furthermore, RNA-seq analysis of *atsnu13* homozygous mutants revealed the abnormal pre-mRNA splicing of a subset of genes that are critical for pathogen resistance. The *atsnu13* mutant affects the abnormal splicing of multiple resistance genes, including *RBOHD* (NADPH respiratory burst oxidase homolog D) and *ALD1* (coding an AGD2-like defense response protein 1), which may have led to the decrease in plant disease resistance. The generation of reactive oxygen species by the NADPH oxidase RBOHD is an important early signaling event that connects PRR- and NLR-mediated immunity. Splicing of ROS-related genes was discovered to be important in *AtSnu13*-associated defense. Our study provides strong evidence that *AtSNU13* can regulate plant immunity in *Arabidopsis thaliana* via inefficient splicing and mis-splicing of several key resistance-related factors.

## Results

### Identification of AtSNU13 in *Arabidopsis thaliana*

**Fig. 1:**
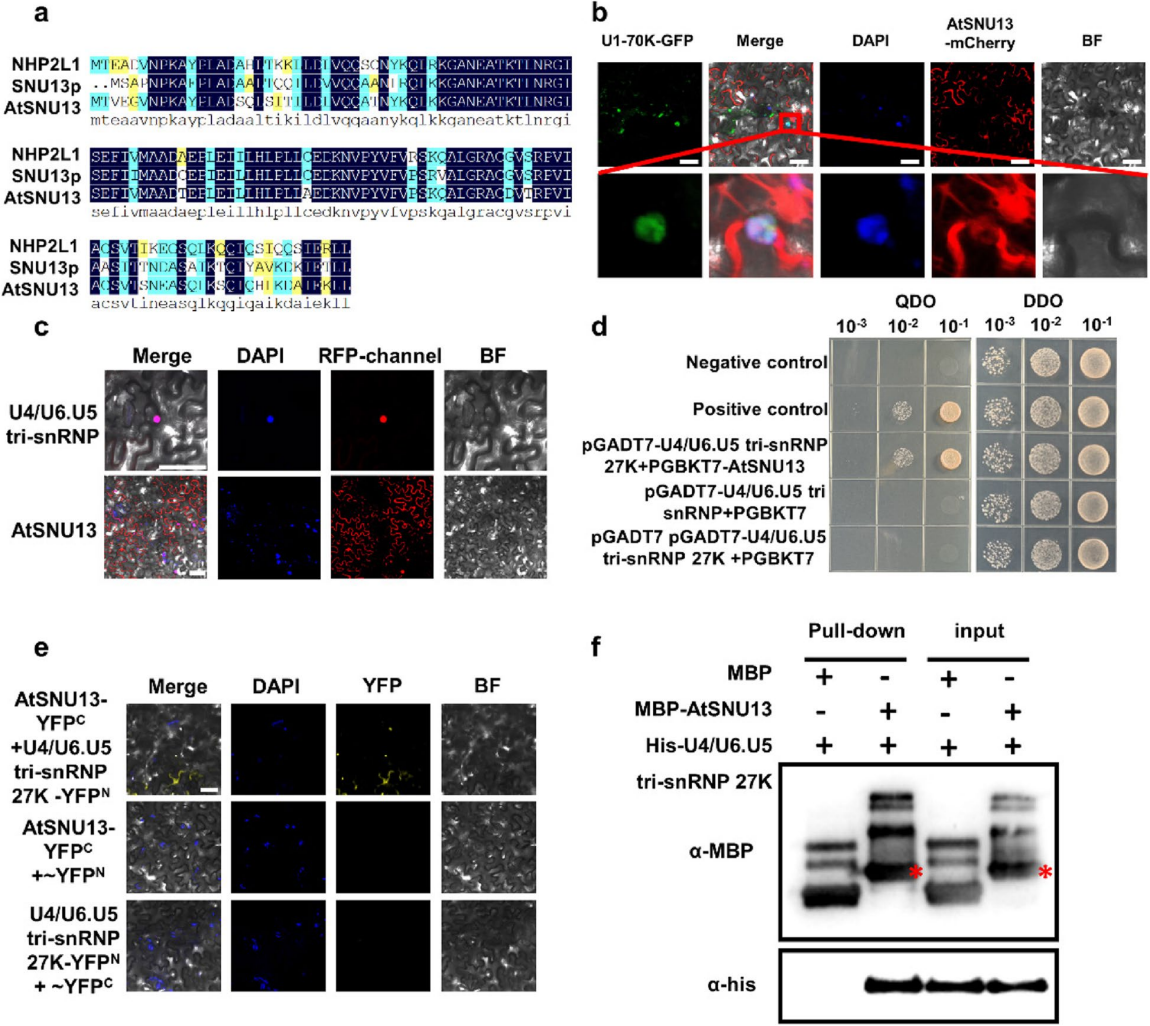
Nuclear localization of AtSNU13 and its physical interaction with U4/U6. U5 tri-snRNP. **a** The alignment of amino acid sequences between the yeast snu13p, human NHP2L1, and AtSNU13. **b** Subcellular localization of AtSNU13 and U1–70 K in *N. benthamiana* leaves and localization of AtSNU13 both in the nucleus and cytoplasm. Its co-localization with U1-70 K in the nucleus; scale bar = 20 μm. **c** Subcellular localization of AtSNU13 and U4/U6. U5 tri-snRNP-specific 27 K in *N. benthamiana* leaves, scale bar = 20 μm. **d** Yeast two-hybrid (Y2H) analysis showing the interaction between AtSNU13 and U4/U6. U5 tri-snRNP. The constructs were co-transformed into the yeast strain Y2HGold cells. The cell growth on selective media SD-Trp-Leu (SD-L-W) and SD-Trp-Leu-His-Ade (SD-L-W–H-Ade). Expression of pGADT7-T + pGBKT7-53 was used as a positive control and pGADT7-T + pGBKT7-lam as a positive control. **e** BiFC analysis showing the interaction between AtSNU13 and U4/U6. U5 tri-snRNP-specific 27 K in *N. benthamiana* leaves; scale bars = 20 μm. **f** In vitro pull-down assay showing the interaction between AtSNU13 and U4/U6. U5 tri-snRNP-specific 27 K. Protein bands of MBP-AtSNU13 are marked by asterisks

We identified more than 30 RNA splicing-related mutants, and it was found that SALK_069227C, a transfer DNA (T-DNA) insertion mutant in *Arabidopsis*, significantly reduced resistance to *Pst* DC3000. In mutants, the T-DNA insertion sites were located in the promoter region of the locus At4g12600. The homozygote mutant was identified by polymerase chain reaction (PCR) analysis using T-DNA-specific primers (Fig. S1a-b). Since the insertion site of SALK_069227C was located in the promoter, we assumed that this mutant might act as a knockdown type mutant to affect the expression of this gene transcription level. The expression of *At4g12600* in the mutant was detected and downregulated compared to the wild-type at the transcriptional level (Additional File 2: Fig. S1c). The phenotypes were observed to determine if mutations in the gene affect plant growth and development (Fig. S2). We found that the gene encoding a putative ortholog of the yeast *SNU13p* is highly conserved between yeast and humans (Fig. 1a), and therefore, we named it *Arabidopsis SNU13* (*AtSNU13*). We further investigated the biological functions of *AtSNU13* and predicted it to encode a 128-amino-acid protein. We analyzed two homologous proteins, yeast SNU13p and human NHP2L1, and identified several conserved residues in the ribosomal_L7Ae domain of AtSNU13, snu13p, and NHP2L1 (Fig. S3a). We performed BLAST analysis using the amino acid sequence against 26 protein databases of 23 representative eukaryotic species at the National Center for Biotechnology Information (NCBI). It revealed that the proteins shared a high degree of similarity between the fungus and the animal (Fig. S3b, Additional File 1: Table S1). The results suggest that AtSNU13 may have functions similar to those of the yeast SNU13 and the human NHP2L1.

### AtSNU13 localizes to the nucleus and interacts with the U4/U6.U5 tri-snRNP-specific 27 K protein

It has been reported that the yeast SNU13 and the human NHP2L1 are small-molecule regulators essential for pre-messenger RNA splicing by associating with U4 snRNA to assemble the U4/U6.U5 tri-snRNP [47]. Therefore, we hypothesized that *AtSNU13* modulated plant resistance against pathogens via RNA splicing. We first investigated the subcellular localization of AtSNU13 in *Arabidopsis*; meanwhile, we used the U1–70 K protein, a spliceosome marker localized to nuclear speckles [48]*.* As anticipated, we found that AtSNU13 localized in the cytoplasm and nucleus, and it co-located with U1–70 K (Fig. 1b). Research has shown that the yeast snu13p is a nucleation factor for U4/U6 RNP assembly [49, 50]. While interacting with snRNP, we focused on its potential role in pre-mRNA splicing. The U4/U6.U5tri-snRNP-specific SR protein, 27 K, is highly homologous to the U1–70 K protein which interacts with the tri-snRNP complex. We hypothesized that the functions of AtSNU13 are related to the U4/U6.U5 tri-snRNP complex, so we generated 35S: *AtSNU13-mCherry* and 35S: U4/U6.U5 tri-snRNP-specific 27 K- *mCherry* before performing a transient expression assay in *N. benthamiana*. The fluorescence emission was detected in the nucleus (Fig. 1c), implying that AtSNU13 colocalizes with U4/U6.U5 tri-snRNP-specific 27 K in the nucleus, revealing the potential correlation between U4/U6.U5 tri-snRNP-specific 27 K and AtSNU13. Moreover, we cloned the full-length cDNA sequence of *AtSNU13* and *U4/U6.U5 tri-snRNP 27 K* into the plasmid pGADT7. Later, we validated this interaction using the yeast two-hybrid (Y2H) assay (Fig. 1d). We also performed the bimolecular fluorescence complementation (BiFC) assay to further confirm this interaction. As shown in Fig. 1e, the recombinant YFP fluorescent signals from AtSNU13-YFP^N–terminal^ and U4/U6.U5 tri-snRNP-specific 27 K- YFP^C–terminal^ showed the interaction between these two in the nucleus. Then, the pull-down assay revealed that AtSNU13 interacted with U4/U6.U5 tri-snRNP-specific 27 K in vitro (Fig. 1f). These results are evident that AtSNU13 interacted with U4/U6.U5 tri-snRNP-specific 27 K and may also have participated in pre-mRNA splicing in *Arabidopsis thaliana*.

### AtSNU13 is required for immunity in *Arabidopsis thaliana*

**Fig. 2:**
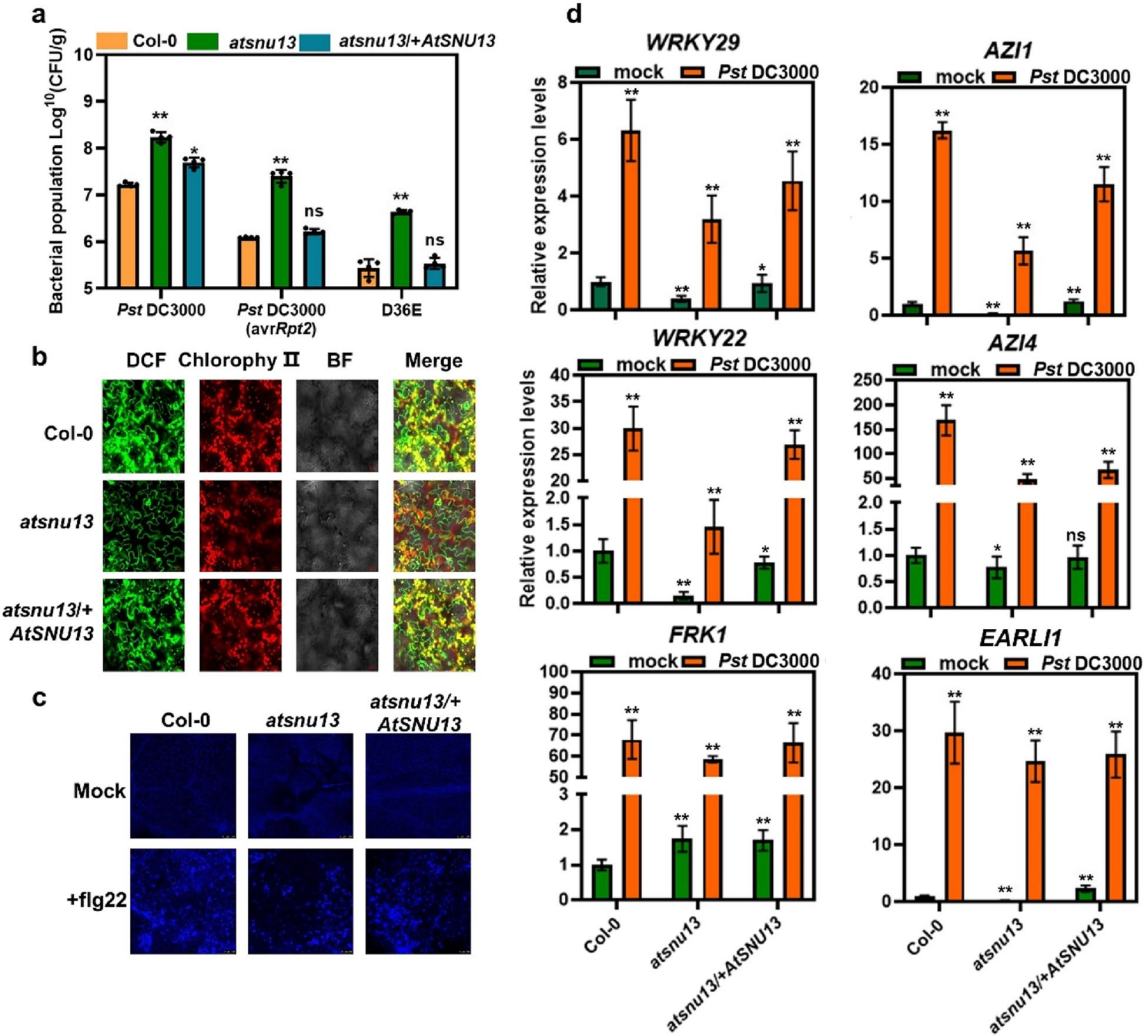
Defects in immunity in *atsnu13* mutant plants. **a** The bacterial populations were determined 2 days post-infiltration (dpi). Two-way ANOVA along with Tukey’s test was performed to compare the means between different treatments (means ± S.E.M, *n* = 4) from leaves of different independent plants. **b** The detection of ROS burst with the fluorescent dye H_2_DCFDA (DCF) in leaves of Col-0, *atsnu13*, and *atsnu13*/ + *AtSNU13* plants at 2 hpt after treatment with flg22. **c** Callose deposition was measured in leaves of Col-0, *atsnu13*, *atsnu13*/ + *AtSNU13* plants at 24 hpt after treatment with flg22 (*n* = 3); scale bars = 100 μm. **d** Canonical marker genes of the flg22-induced PTI pathway, including *WRKY22*, *WRKY29*, and *FRK1*, and genes associated with ETI-mediated immune boosting, including *AZI1*, *EARLI1*, and *AZI4* in Col-0, *atsnu13*, and *atsnu13/* + *AtSNU13* plants after treatment with *Pst* DC3000 inocula and mock; data represent means ± S.E.M (*n* = 3; *P* < 0.01; Student’s *t*-test)

We cloned the *AtSNU13* genome sequence (2913 bp) to confirm if the mutant was responsible for the plant resistance against the pathogen, which contained the predicted promoter region of 2000 bp and all introns and exons, to build a pro*AtSNU13*: *AtSNU13* genomic-*mCherry* expression vector. This construct was introduced into *atsnu13* mutants using *Agrobacterium*-mediated infiltration to generate *AtSNU13 g-mCherry* transgenic plants. We examined the expression of *AtSNU13* at the transcriptional level by both RT-PCR and RT-qPCR (Fig. S4a-b). To further confirm their role in plant immunity, bacterial populations were determined 3 days post-infiltration in wild-type, *atsnu13*, and *atsnu13/* + *AtSNU13 g*-*mCherry* plants. We also evaluated the defense against *Pst* DC3000 (avr*Rpt2*) and D36E and found that *atsnu13* exhibited compromised resistance to *Pst* DC3000(avr*Rpt2*) and D36E, suggesting that the *atsnu13* compromised the resistance via both regulating ETI and PTI pathways. In contrast, *atsnu13/* + *AtSNU13* was recovered from the resistant plants (Fig. 2a). Due to the resistant defect phenotype in *atsnu13*, we detected the expression level of *AtSNU13* after inoculating with *Pst* DC3000, *Pst* DC3000 (avr*Rpt2*), and D36E in wild-type plants. As Fig. S5 shows, the expression of *AtSNU13* was the lowest at 2 hpt after inoculation, and then it was gradually upregulated at 4–8 hpt after inoculation. The result shows that *AtSNU13* seems not specifically induced by the pathogen.

We detected the immune responses, such as the reactive oxygen species (ROS) burst, and callose deposition in wild-type, *atsnu13*, and *atsnu13/* + *AtSNU13* g-*mCherry* plants to further investigate the *AtSNU13-*mediated plant immunity. PTI and ETI have been linked to the production of ROS, a key function of the immune system that has been proposed to act as the first line of defense, killing pathogens, as well as signaling molecules that further activate immune responses [51]. Since flg22 is a classical effector that could induce the PTI defense in plants [52], we used it to trigger the PTI response and observe the difference in the key PTI response between wild-type and *atsnu13* plants. First, we monitored ROS burst by using the fluorescent dye H_2_DCFDA (Fig. 2b). The results showed that the *atsnu13* mutant exhibited a defective phenotype of PTI-triggered ROS. To further detect the accumulation of H_2_O_2_ and O^2–^ in wild-type and *atsnu13*, we conducted an assay with the 3,3-diaminobenzidine (DAB) and tetranitro blue tetrazolium chloride (NBT) staining solution to detect the ROS burst in 10-days-old *atsnu13* and wild-type plants treated with flg22 (Fig. S6). The staining intensity observed in Fig. S6b indicates that *atsnu13* could not elicit a similar flg22-indued PTI response in wild-type plants. Because callose deposition is a key PTI response, we detected it with an aniline blue solution. It was discovered that the fluorescence signal intensity was lower in *atsnu13* plants compared to wild-type plants (Fig. 2c, Fig. S7). Above all, we discovered that *atsnu13* had a weakened PTI response compared to the wild-type, and the *atsnu13* mutant reduced plant pathogen resistance, which could be mediated by both PTI and ETI signaling pathways.

It has been reported that the expression of defense-response genes is accompanied by pathogen infection [1, 53, 54]. Expression analyses were performed using RT-qPCR to determine whether the transcription of defense-related genes was affected in the *atsnu13* mutant. We found that several canonical marker genes of the flg22-induced PTI pathway, including *WRKY22*, *WRKY29*, and *FRK1*, and genes related to ETI-mediated immunity boosting (*AZI1*, *EARLI1*, and *AZI4*) [6], were significantly downregulated in *atsnu13* (Fig. 2d).

### AtSNU13 mutation causes a major pre-mRNA splicing defect

**Fig. 3:**
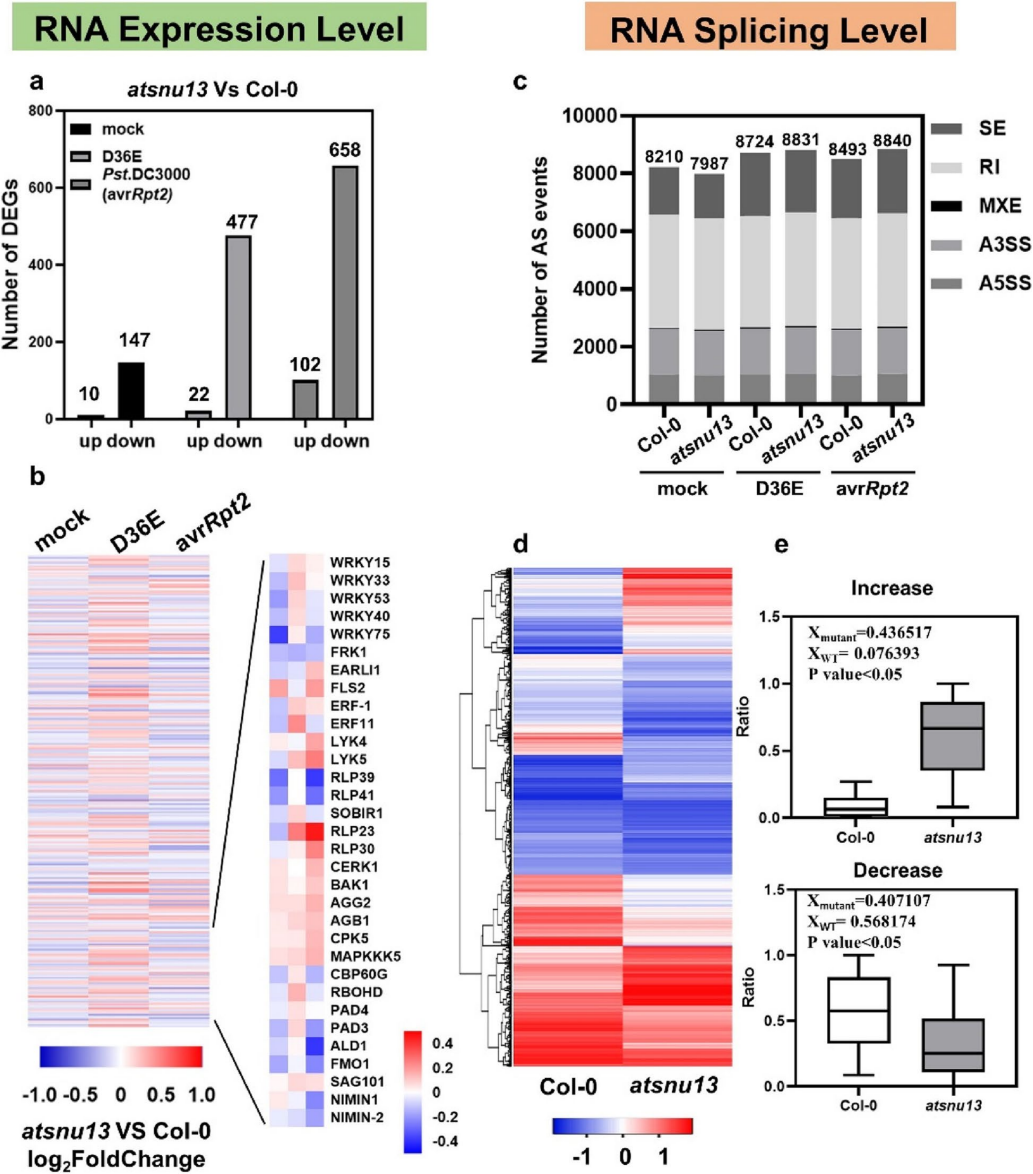
RNA-seq analysis of the *Arabidopsis thaliana* Col-0 and *atsnu13* mutants. **a** Summary of differentially expressed genes in *atsnu13*; the left column shows representative upregulated differentially expressed genes, while the right column shows representative downregulated differentially expressed genes. **b** DEGs related to plant immunity; the numerical values for the white-to-black gradient bar represent the log2-fold change values in *atsnu13* compared to those in Col-0. **c** Statistical chart of AS events; ES, exon skipping; IR, intron retention; A5SS; A3SS in Col-0 and *atsnu13* after D36E and avr*Rpt2* treatment. **d** Heat map of differential pre-mRNA splicing profiling in both the Col-0 and *atsnu13* plants based on the exon-inclusion levels detected by RNA-seq analysis. **e**, **f** Box plots showing the decrease and increase in the ratio (PSI/PIR) of splicing events of differential alternative splicing genes in WT and *atsnu13* plants

Considering that *AtSNU13* encoded the ortholog of the yeast splicing factor *Snu13p* and interacted with the core protein component of spliceosome, we reasoned that *AtSNU13* is likely to be involved in pre-mRNA splicing. To investigate the functional role of *AtSNU13* in mRNA metabolism in the immune system, we harvested leaves of *atsnu13* mutant and wild-type plants at 0 hpt and 2 hpt after infection with D36E and *Pst* DC3000 (avr*Rpt2*) and subjected them to ultra-high-throughput RNA sequencing (RNA-seq; Illumina). A total of 18 samples were tested using the BGISEQ500 platform, and each sample produced an average of 11.31G of data. The samples were divided into nine groups with three biological replicates. The average comparison rate of the *Arabidopsis* genome and gene sets was 93.58% and 88.10% respectively. A total of 23,701 genes were detected (Additional File 1: Table S2-S4). Sequence reads were mapped to exonic and intronic regions and also to the exon–intron junctions of all genes in the *Arabidopsis* genome. The pre-mRNA splicing patterns in wild-type and *atsnu13* mutant plants were compared. The results showed that there were 157 differentially expressed genes (|log_2_FC|≥ 1, *P* ≤ 0.05), with 10 upregulated genes and 147 downregulated genes in *atsnu13* mutant plants compared to the wild-type (Col-0) (Fig. 3a). After D36E and av*Rpt2* treatment, there were 22 and 102 upregulated and 477 and 658 downregulated genes, respectively in *atsnu13* plants (Fig. 3a, Additional File 1: Table S5)*.* Moreover, DEG expression significantly increased after pathogen infection. Downregulated genes accounted for the majority of genes in *atsnu13* compared to wild-type plants, indicating that *AtSNU13* plays a role in regulating gene expression. A heatmap of the differentially expressed genes revealed that *atsnu13* plants had different expression patterns than wild-type plants (Fig. 3b). Gene ontology analyses showed the presence of genes with altered expression patterns in *atsnu13*, including chitin response, defense response, oxidative stress response, systemic acquired resistance (SAR), and phosphorylation (Fig. S8). We noted changes in the expression of a large number of genes associated with plant immunity, such as *WRKY-FRK1* genes (*WRKY15*, *WRKY33*, *WRKY53*, *WRKY40*, *WRKY75*, *FRK1*), and *RLK-LYK5-RLP* pathway genes (*FLS2*, *ERF-1*, *LYK5*, *LYK5*, *RLP39*, *SOBIR1*, *CERK1*, *BAK1*, *AGG2*, *AGB1*, *CPK5*, *MAPKKK5*, *CBP60G*) in plants treated with mock, D36E, and avr*Rpt2* (Fig. 3b, Additional File 1: Table S6). The results were verified using RT-qPCR by analyzing the expression of these differentially expressed genes in wild-type and *atsnu13* plants treated with mock, D36E, and avrRpt2*.* The expression of the defense-related genes, including *WRKY 40*, *WRKY 60*, *FMO1*, *ALD1*, *CPK5*, and *CERK1* was downregulated in *atsnu13* plants treated with mock, D36E, and avr*Rpt2* (Fig. S9). These results suggest that *atsnu13* is essential for plant immunity.

We took *AtSNU13* as a splicing factor that may be involved in mRNA splicing, and we performed the AS analysis in wild-type and *atsnu13* plants. RNA-seq data showed that there were 8210, 8724, and 8493 AS-related genes in wild-type plants treated with mock, D36E, and avr*Rpt2*, respectively, while 7987, 8831, and 8840 genes were found in *atsnu13* plants who received the treatment with mock, D36E, and avr*Rpt2*, respectively (Fig. 3c, Additional File 1: Table S7). Additionally, we supplemented the raw data in the transcriptome (Additional File 1: Table S7), which explained the total number of splicing events and showed changes in the number of splicing events in the mutant compared to the wild-type. This suggested that the *atsnu13* caused fewer AS events, while pathogens attack, showed more AS events, which indicates that AtSNU13 affects the gene splicing of plants. To further demonstrate the substantive differences between Col-0 and the *atsnu13* mutant, the different alternative splicing events between Col-0 and the *atsnu13* mutant in 3 different treatments detected by the RNA-seq lump together in Table S8 (Additional File 1). We also calculated the changes in the inclusion ratio in wild-type and *atsnu13* mutants, and as the heat map shows, the splicing patterns in *atsnu13* were altered (Fig. 3d). Box plots of PSI and PIR of alternative splicing differential genes in Col-0 and *atsnu13* and the box plots also showed the evolution of splicing efficiency in *atsnu13* (Fig. 3). Then, according to the transcriptome statistics of the alternative splicing events in *atsnu13*, we designed three primers to verify the authenticity of the splicing events according to the location of the splicing events (Additional File 1: Table S8). We used RT-qPCR to investigate the mis-splicing transcripts to validate the results of the RNA-seq analysis of differential alternative splicing genes. After inoculation, the *atsnu13* mutant exhibited an increased frequency of AS events, suggesting a potential association between pathogen infection and the generation of AS events in plants. These results demonstrate the functional role of *AtSNU13* in the splicing of genes and the possible association with the transcription expression of target genes. In conclusion, *AtSNU13* is involved in mRNA splicing.

### AtSNU13 is involved in pre-mRNA splicing of defense-related genes to function in plant immunity

**Fig. 4:**
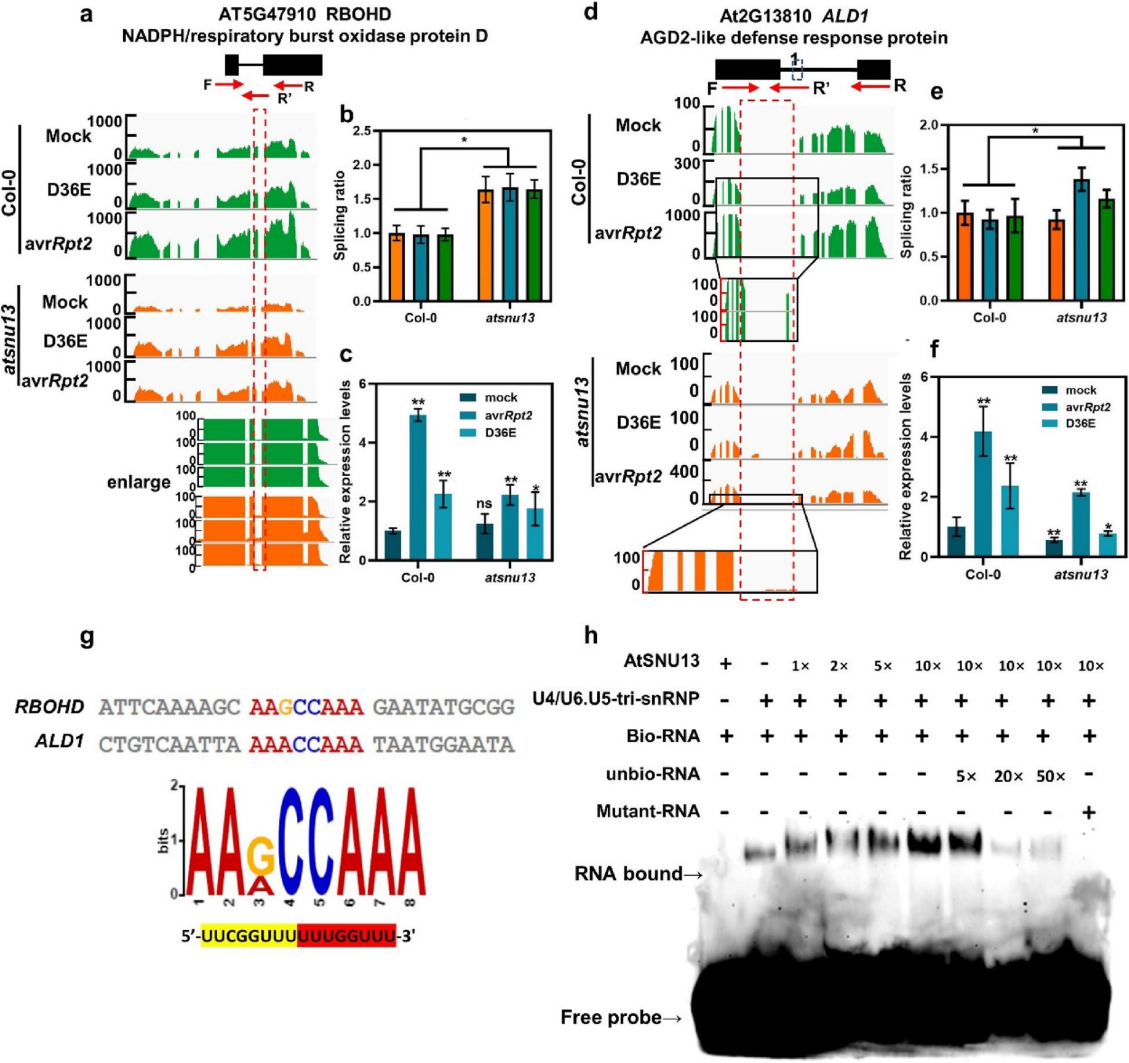
Mis-splicing of defense-related genes in *atsnu13*. **a** Wiggle plots of RNA-seq data in Col-0 and *atsnu13* plants with the *RBOHD* (At5 g47910) gene track shown at the bottom. **b**, **e** RNA-seq and qRT-PCR analyses for validation of abnormal splicing of *RBOHD*; the non-spliced and spliced RNA levels were tested with F/R and F/R′ primers, respectively. Splicing efficiency was determined by normalizing the abundance of the spliced transcripts to that of the non-spliced transcripts. Data represent means ± S.E.M (*n* = 3; * *P* < 0.05; Student’s *t*-test); F, forward primer; R, reverse primer. **c** The transcriptional expression of *RBOHD* in plants treated with mock, avr*Rpt2*, and D36E; data represent means ± S.E.M (*n* = 3; *P* < 0.01; Student’s *t*-test). **d** Wiggle plots of RNA-seq data in Col-0 and *atsnu13* plants with the *ALD1* (At2 g13180) gene track shown at the bottom. **f** The transcriptional expression of *ALD1* in plants treated with mock, avr*Rpt2*, and D36E; data represent means ± SD (*n* = 3; *P* < 0.01; Student’s *t*-test). **g** Predict motif “5′-AAGCCAAA-3′” in *RBOHD* and *ALD1.* **h** EMSA assay confirming the in vitro binding of U4/U6. U5 tri-snRNP-specific 27 K to the predicted motif and also its enhanced interaction with AtSNU13 in a dose-dependent manner

The *atsnu13* mutants were identified based on the defects in plant immunity in plants, and we found several genes undergo abnormal splicing at different splice sites in *atsnu13* mutant (Fig. S10), among which several genes were associated with plant immunity, including a ROS burst-related gene *RBOHD* and a key PTI component gene ALD1 (AGD2-like defense response protein 1) (Fig. 4b, 4e, S10). Hence, we investigated the pre-mRNA splicing using primers and found changes in the splicing profile in *atsnu13* during infection with D36E and avr*Rpt2* (Fig. 4b). The transcriptional expression of *RBOHD* was lower in the *atsnu13* mutant than in wild-type plants infected with mock, D36E, and avr*Rpt2* (Fig. 4c). *ALD1*, another mis-spliced gene, is linked to systemic acquired resistance (SAR) and is involved in both local and whole-plant immune signaling. During D36E and avr*Rpt2* infection, the splicing ratio of *ALD1* in intron 2 was higher in *atsnu13* than in wild-type plants (Fig. 4d, e). In contrast, the expression of the gene was lower in *atsnu13* than in wild-type plants (Fig. 4f). The general trends of expression of these two genes (Fig. 4C, F) are the same in Col-0 and mutant, suggesting that *AtSNU13* affecting the mis-splicing of genes, the transcriptional expression level of genes was also reduced at the same time. These findings suggest that AtSNU13 regulates plant immunity via pre-mRNA splicing of defense-related genes.

### AtSNU13 promotes the interaction between U4/U6.U5 tri-snRNP-specific 27 K and the *cis*-element in target mRNAs to regulate the RNA splicing

AtSNU13 interacted with the U4/U6.U5 tri-snRNP-specific 27 K protein, which is a novel SR protein with an snRNP 27 K domain that could bind to mRNA [55]. Based on this observation, we hypothesized that AtSNU13 might interfere with both the binding of U4/U6.U5 tri-snRNP-specific 27 K to the target mRNA and its recognition. This interference can result in defects in the immune system. We examined the interaction between U4/U6.U5 tri-snRNP-specific 27 K and the target mRNA. First, we found the target genes, *RBOHD* and *ALD1*, that commonly contain the motif “5′-AAGCCAAA-3′” (Fig. 4g). Next, to verify the interaction of U4/U6.U5 tri-snRNP-specific 27 K protein with the cis-element, we carried out the electrophoretic mobility shift assay (EMSA) by using the purified His- AtSNU13 fusion protein and the U4/U6.U5 tri-snRNP-specific 27 K-MBP fusion protein (Fig. S11). The results showed that the single U4/U6.U5 tri-snRNP-specific 27 K protein could bind to motifs, but the single AtSNU13 could not bind to motifs, and the binding of the protein pairs U4/U6.U5 tri-snRNP and AtSNU13 to RNA significantly increased (Fig. 4h).

### AtSNU13 is required for coupling between transcription and RNA splicing in plant immunity

**Fig. 5:**
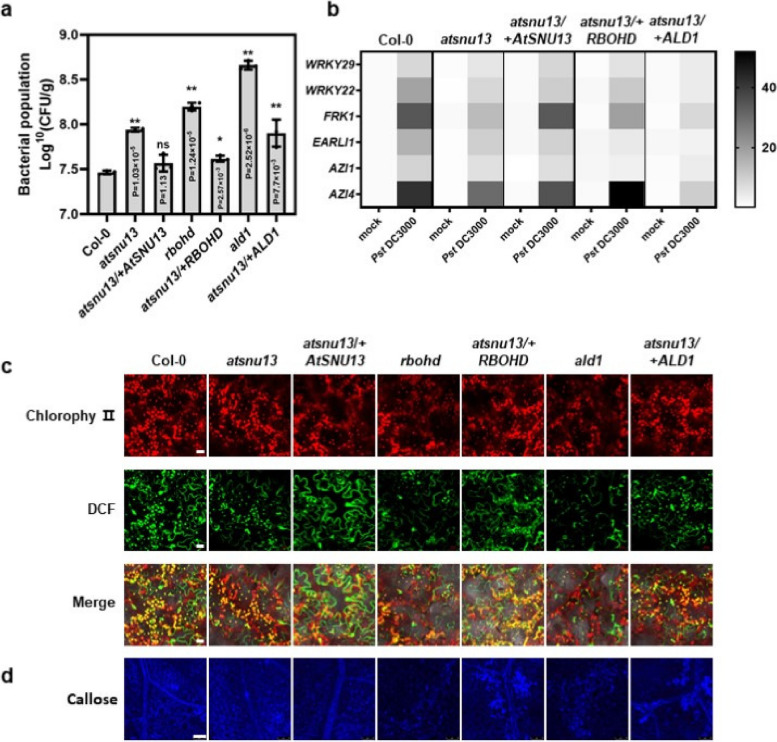
Enhanced disease resistance and transcriptional expression in *atsnu13*/ + *RBOHD* and *atsnu13*/ + *ALD1* transgenic plant lines. **a** The bacterial populations were determined 2 days post-infiltration (dpi) in Col-0, *atsnu13*, *atsnu13*/ + *AtSNU13, rbohd, atsnu13/* + *RBOHD*, *ald1* and *atsnu13/* + *ALD1* plants. Two-way ANOVA, along with Tukey’s test, was performed. Data represent means ± S.E.M (*n* = 3 biologically independent samples). **b** Canonical marker genes of the flg22-induced PTI pathway, including *WRKY22*, *WRKY29*, and *FRK1*, and genes related to ETI-mediated immune boosting, including *AZI1*, *EARLI1*, and *AZI4* in Col-0, *atsnu13*, *atsnu13*/ + *AtSNU13, rbohd, atsnu13/* + *RBOHD*, *ald1* and *atsnu13/* + *ALD1* plants after treatment with *Pst* DC3000, *Pst* DC3000(avr*Rpt2*), and D36E inocula and mock*.* Data represent means ± S.E.M (*n* = 3; *P* < 0.01; Student’s *t*-test). **c** Detection of ROS burst with the fluorescent dye DCF-DA (DCF) in leaves of Col-0, *atsnu13*, *atsnu13/* + *AtSNU13*, *rbohd, atsnu13/* + *RBOHD*, *ald1*, and *atsnu13/* + *ALD1* plants at 2 hpt after treatment with flg22; scale bars = 20 μm. **d** Callose deposition was measured in leaves of Col-0, *atsnu13*, *atsnu13*/ + *AtSNU13*, *rbohd, atsnu13/* + *RBOHD*, *ald1*, and *atsnu13/* + *ALD1* plants at 24 hpt after treatment with flg22 (*n* = 3); scale bars = 100 μm

We further examined how the abnormal splicing mediated by *RBOHD* and *ALD1* led to defects in plant immunity. For this purpose, we cloned the predicted 1800-bp promoter region into the coding sequence (2766 bp) of *RBOHD* and the coding sequence (1371 bp) of *ALD1* to build the pro-*RBOHD*: *RBOHD*^CDS^: GFP and the pro*-ALD1*: *ALD1*^CDS^: GFP expression vectors, respectively. These constructs were then introduced into the *atsnu13* mutants via agrobacterium-mediated infiltration to generate *RBOHD*-*GFP* transgenic plants (*atsnu13*/ + *RBOHD*-*GFP*) and *ALD1*-*GFP* transgenic plants (*atsnu13*/ + *ALD1*-*GFP*), respectively. The *atsnu13/* + *RBOHD-GFP* transgenic plants showed greater resistance than the *atsnu13* mutant but a lower resistance than *atsnu13/* + *AtSNU13*. Additionally, the *rbohd* mutant plants exhibited lower resistance. The results suggest that *atsnu13*-mediated defects in immunity are most likely caused by the mis-splicing of *RBOHD*. It is worth noting that *ALD1*-GFP transgenic plants did not show full recovery of resistance, while the *ald1* mutant exhibited susceptibility to the pathogens (Fig. 5a). These results indicate that although the mis-splicing of *ALD1* has a certain effect on the resistance of Arabidopsis, its effect is weaker than that of mis-splicing of *RBOHD*.

We found that the expression of PTI and ETI-related genes in the *atsnu13* mutant decreased compared to the wild type during pathogen infection (Fig. 2d). These genes included canonical marker genes of the flg22-induced PTI pathway, including *WRKY22*, *WRKY29*, and *FRK1*, and ETI-mediated genes involved in immune boosting, including *AZI1*, *EARLI1*, and *AZI4.* This observation was further verified in wild-type, *atsnu13*, *atsnu13*/ + *AtSNU13*, *atsnu13*/ + *RBOHD*, and *atsnu13*/ + *ALD1* plants (Fig. 5b)*.* The transcriptional expression in *atsnu13*/ + *AtSNU13* and *atsnu13*/ + *RBOHD* partially recovered compared to that in the *atsnu13* mutant, suggesting that the mis-splicing of *RBOHD* potentially co-transcriptionally mediated the transcriptional expression levels. However, the *atsnu13*/ + *ALD1* did not recover the expression of defense-related genes completely. This result implies that the mis-splicing of *ALD1* caused by *atsnu13* has a very weak effect on plant immunity. We believe that the influence of splicing factor *AtSNU13* on plant immunity is not limited to the wrong splicing of one or two certain genes but should be caused by the splicing differences of an assembly of genes.

We used an elaborate molecular assay to evaluate immune responses in transgenic plants to confirm the mechanism of *AtSNU13* mediating aberrant splicing of *RBOHD* that compromises PAMP-triggered immunity responses. First, we monitored ROS burst by the fluorescent dye H_2_DCFDA in wild-type, *atsnu13*, *atsnu13*/ + *AtSNU13*, *atsnu13*/ + *RBOHD*, *rbohd, atsnu13*/ + *ALD1*, and *ald1* plants treated with flg22. The fluorescent intensity of leaves in *atsnu13*/ + *RBOHD* and *atsnu13*/ + *ALD1* plants recovered compared to that in *atsnu13*. In contrast, the *rbohd* and *ald1* mutant plants exhibited lower intensity (Fig. 5c). Furthermore, to examine whether the same or different amounts of H_2_O_2_ and O^2–^ accumulated in *atsnu13*/ + *RBOHD* compared to those in *atsnu13*, DAB and NBT staining assays were conducted; the intense brown (for DAB) and blue (for NBT) spots observed on leaves were the same as those for DCF assay (Fig. S12). We also detected the callose deposition with staining of 35-day-old wild-type, *atsnu13*, *atsnu13*/ + *AtSNU13*, *atsnu13*/ + *RBOHD*, and *rbohd* plants. The *atsnu13*/ + *RBOHD* showed a higher fluorescence intensity than *rbohd* but lower than *atsnu13*/ + *AtSNU13* (Fig. 5d, Fig. S12), implying that by mis-splicing of *RBOHD* and *ALD1*, *atsnu13* compromised early immune response partially*.* Based on our findings, we suggest that *AtSNU13* mediating the mis-splicing of *RBOHD* plays a role in PAMP-triggered immunity in *Arabidopsis thaliana.*

**Fig. 6:**
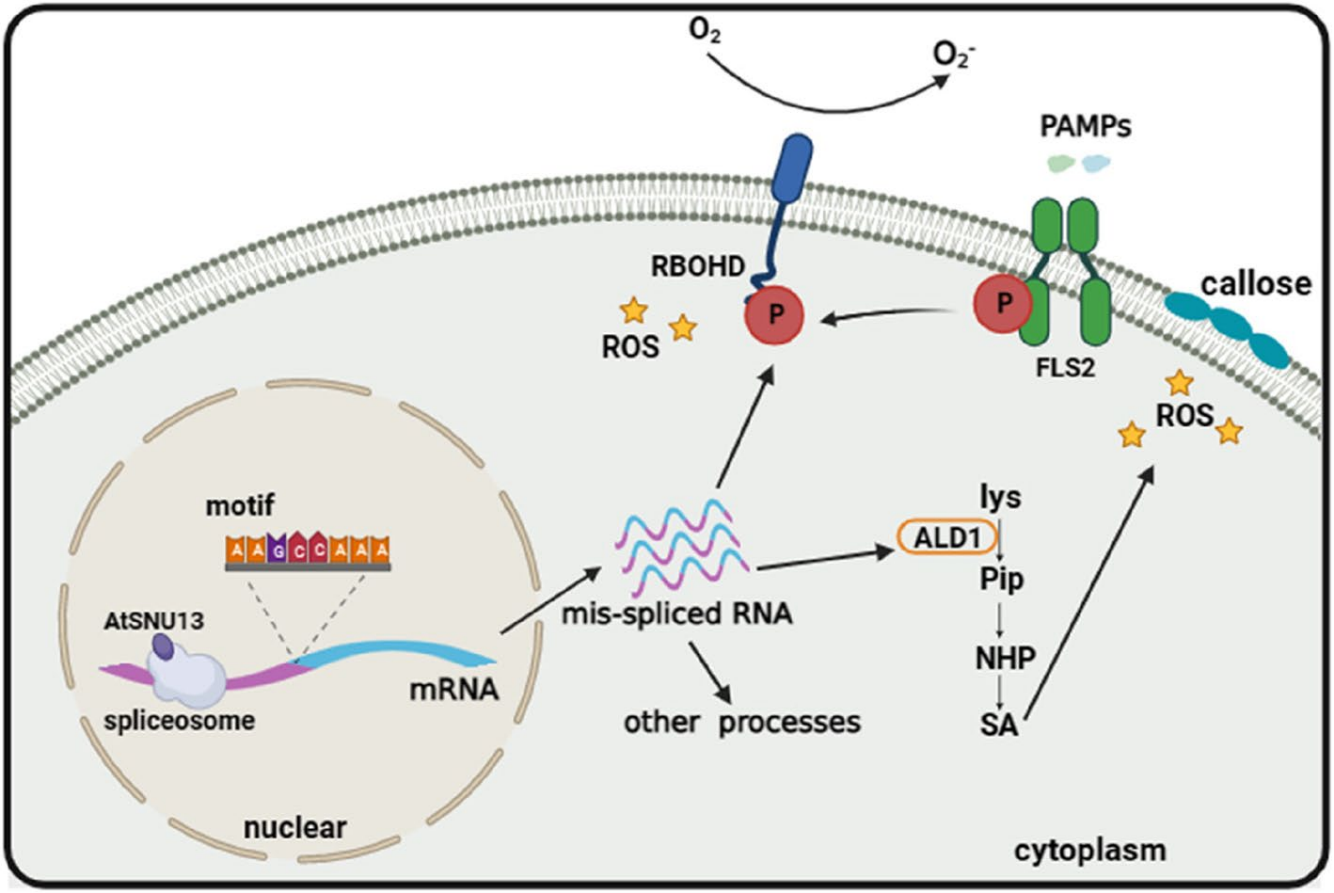
A schematic model depicting the roles of AtSNU13 in plant immune responses mediated by signaling pathways. AtSNU13 interacted with the spliceosome component to regulate PAMP-triggered immunity in *Arabidopsis* by disturbing the normal splicing of *RBOHD* and *ALD1.* This phenomenon resulted in the regulation of the ROS burst and other key immune signaling pathways. Created with BioRender.com

We proposed a dynamic model that AtSNU13 binds to the component of the spliceosome, generating sets of mis-spliced isoforms of PAMP-associated genes and result to changes in the transcript levels of genes in response to pathogens to regulate downstream immune signaling pathways, including RBOHD-mediated ROS burst. NADPH oxidase RBOHD is the critical early signaling in plant innate immunity and produces reactive oxygen in the process of both PTI and ETI response. The splicing of RBOHD leads to a decrease in the levels of reactive oxygen, thereby reducing the plant’s immune response. The ALD1-mediated SA pathway in resistance, while the ald1 gene controls the synthesis of Pipecolic acid induced by pathogenic bacteria to enhance SAR in plants, by increasing NO and ROS levels [56]. Even though we found that the alteration in the splicing efficiency of ald1 does not directly impact the weakening of plant immunity caused by *atsnu13*, its splicing efficiency may influence the transmission of systemic acquired resistance (SAR). Ultimately, the occurrence of splicing events mediated by *AtSNU13* may play a role in other aspects of plant immunity (Fig. 6).

## Discussion

RNA splicing is an important post-transcriptional mechanism in eukaryotic organisms. In *Arabidopsis*, about 430 spliceosomal factors have been identified or predicted [57]. Furthermore, the identified splicing-related proteins are mainly based on the conservation of certain functionally important domains. So far, quite a few genetic studies have identified the homologs of yeast or human putative RNA-binding factors in *Arabidopsis* and demonstrated its role in pre-mRNA splicing [58–61]. Hence, we explored this idea by identifying AtSNU13 in Arabidopsis, the homologous gene of the yeast Snu13p and human NHP2L1, as a component of U4/U6.U5 tri-snRNP. Interestingly, the amino acid sequence alignment of AtSNU13, yeast Snu13p, and human NHP2L1 showed that AtSNU13 might have the same function as yeast Snu13p and human NHP2L1 (Fig. 1a). The highly conserved L7ae domain was discovered through phylogenetic analysis (Fig. S3), indicating that the L7ae is an ancient protein with an evolutionarily conserved function. Recently, a study showed that SNU13 functions in the salt tolerance mechanisms [62]. In contrast, human NHP2L binds to U4 snRNA during spliceosome assembly, is involved in RNA splicing, and mediates the development of human tumors [44]. Our study revealed that the defect of the AtSNU13 function causes susceptibility to pathogens (Fig. 2a, b). The data show that SNU13 has conserved functions and plays a critical role in the evolution of unicellular organisms into multicellular organisms.

Research has shown that the core small nuclear RNA (snRNA) ribonucleoproteins (snRNP), the retention and splicing complex (RES), serine/arginine-rich (SR) proteins, heterogeneous nuclear ribonucleoproteins (hnRNP), and a large number of RNA-binding proteins and associated regulatory factors are involved in pre-mRNA splicing in plants. For example, a conserved pre-mRNA splicing factor PRP31 was identified to be involved in the formation of the U4/U6.U5 snRNP complex in fungi and animals [62]. The serine/arginine-rich (SR) protein is an important regulator of constitutive and/or alternative splicing and other aspects of mRNA metabolism [63]. *Arabidopsis thaliana* BUD13 (AtBUD13), growth, development, and splicing 1 (GDS1), and DAWDLE (DDL) are the counterparts of the yeast RES complex subunits mediating pre-mRNA splicing of multiple genes [64]. We observed that AtSNU13, a splicing-related factor, directly interacts with U4/U6.U5 tri-snRNP (Fig. 1b–f) in some cases. Thus, AtSNU13 seems to be involved in the assembly and activation of the spliceosome, which is catalyzed by U4/U6.U5 tri-snRNP. The RNA-seq analyses showed a specific subset different from AS events, and the total number of AS events differed between *atsnu13* and Col-0 (Fig. 3c). We also identified 1808 differential pre-mRNA splicing profiling in *atsnu13* compared to the Col-0 (Fig. 3d, e). We assumed the *AtSNU13* function in the pre-mRNA splicing of a subset of genes, which functionally conserved in its homologous gene. We hypothesize that AtSNU13, as a U4/U6.U5 tri-snRNP, influences splicing and AS of several resistance-related genes and that the result is most likely a combination of many factors that affect several aspects of pathogen response. It is worth noting that its yeast ortholog genes are known as the RNA-binding protein SNU13p, which binds U4 snRNA from the spliceosome as well as box C/D snoRNAs from the pre-ribosomal RNA processing machinery to induce the assembly of each ribonucleoprotein complex [65–67]. In this study, we focused more on its function in pre-mRNA splicing, while we also want to consider looking at rRNA processing, since this may be a factor as well. We detected the total RNA in the Col-0, *atsnu13* mutant, and *atsnu13*/ + *AtSNU13* lines (Fig. S13), which includes 28 s rRNA, 18 s rRNA, and 5 s rRNA. The results showed that the 28 s rRNA and 18 s rRNA decreased in the *atsnu13* mutant compared to the Col-0, while they are recovered in the *atsnu13*/ + *AtSNU13* lines. Researchers identified snoRNAs and predicted to guide trypanosome-specific rRNA cleavages [68], so the function in pre-ribosomal RNA processing machinery needs further research.

In the absence of splicing factors, abnormal splicing events occur in organisms, which results in distinct phenotypes. The U1 snRNP component RBP45d regulated temperature-responsive flowering in Arabidopsis [27]. The RNA-binding protein FgRbp1 regulated specific pre-mRNA splicing via interacting with U2AF23 in Fusarium [69]*.* Substantially, some splicing factors regulate the plant transcriptome under normal growth conditions [70, 71], and the systematic recognition of splicing factors and their target genes is a great concern. A fair number of studies have shown that gene splicing is influenced by different plant stresses, including salt stress [28] and heat shock [72], but there is little evidence that pre-mRNA splicing has a role in plant immunity. After several years of intensified research on plant–microbe interactions, the long-term observed similar immune signaling cascades and downstream defense outputs overlapping between PTI and ETI gradually became the point of focus. Many genes undergo RNA splicing during the plant immunity process. For example, the dynamic regulation of pep-induced immunity occurred through post-translational control of splicing of the *CPK28* transcript [73]. Apart from this, in the long-term evolution, the splicing mechanism y regulates the plant–microbe interactions to challenge plant immunity, suggesting the functional role of mRNA splicing in effector-triggered immunity. PsAvr3c, a virulence effector identified from oomycete plant pathogen, has developed a strategy that reprograms host pre-mRNA splicing to invade host immune systems [74]. Besides, recent analyses of effectors modulating genome-wide alternative splicing of host mRNAs to reprogram plant immunity highlight the significance of plant–microbe interaction at different levels of alternative splicing [75]. Our findings suggested an intriguing post-transcriptional mechanism by which *AtSNU13* could regulate the splicing of defense-related genes to suppress plant PAMP-induced immunity. However, we discovered that the transcripts were different in plants (Fig. 4a, d and Fig. S10), implying that RNA splicing reprogramming may differ during ETI and PTI responses. Our results suggest that the complex AS process, as an essential post-transcriptional mechanism, plays an important role in PAMP-triggered immunity, and there are differences between ETI and PTI responses. PTI and ETI activation in plants induces transcriptional reprogramming [6] and differentially accumulated metabolites [76]. The difference in dynamic reprogramming of pre-mRNA splicing in two modes of plant immunity is still unknown. In the future, the real challenge will be to uncover the underlying mechanisms of mediating the spliceosome component and other splicing factors during plant immunity in layers of RNA splicing. Meanwhile, spliceosome dysfunction is likely to deregulate pathogen response on many levels via inefficient splicing and mis-splicing of several key resistance-related factors. We demonstrate that RNA splicing modulates plant immunity during plant–microbe is complex, and it is difficult to modulate plant immunity through a specific and unique mechanism mediated by one or only a few factors.

Many splicing factors are involved in the dynamic regulation of the plant transcriptome in response to biotic and abiotic stresses. The regulation of AS in response to pathogens is carried out by alternative isoforms and co-transcriptional processes of the main defense factors. We found *atsnu13* modulated plant immunity by both the RNA splicing process and the transcriptional expression. Several genes related to defense have downregulated expression in mRNA levels (Fig. 3a, b). A splicing defect usually alters the level of mature mRNAs in splicing mutants, but response to transcription is also possible, though this was not tested in this study. Our research demonstrates alterations in both disease-resistance gene expression and splicing levels in the *atsnu13* mutant. We consider the changes in transcription and splicing levels mediated by *atsnu13* to be linked. There are two proposed mechanisms for this coupling: interactions between transcription and splicing machinery (recruitment model) or the rate of polymerase elongation, which determines the occurrence of co-transcriptional processes and the extent of AS events. The modulation of plant immune signaling can have cascading effects, which could explain the series of changes in gene expression levels observed in the mutant. The interaction of AS and transcription is critical to plant immunity regulation via splicing. However, more research is needed to determine how co-transcriptional splicing is affected by gene expression levels.

## Conclusion

In our study, we report that a splicing factor conserved in eukaryotes, AtSNU13, is involved in mediating plant defense against pathogens. The interaction between AtSNU13 and the spliceosome component U4/U6.U5 tri-snRNP-specific 27 K was verified by in vivo and in vitro interactions. Genetic tests have verified that AtSNU13 is involved in plant defense, especially affecting classical PTI response. RNA-seq data showed that atsnu13 affects transcriptional expression and splicing of genes in plants. Splicing gene analysis showed that the defense-related genes RBOHD and ALD1 were mis-spliced in the mutant compared with the wild type. The ATsnu13-compromised disease resistance and disease response can be restored to some extent by replacing normal transcripts in atsnu13 mutants. These findings have important implications for the role of post-transcriptional modifications in plant defense.

## Materials and methods

### Plant materials and growth conditions

*Arabidopsis* ecotype Columbia (Col-0) was used as the wild-type control and to generate transgenic plants. Seeds of *atsnu13* mutants were obtained from the Arabidopsis Biological Resource Center, OH, USA (https://abrc.osu.edu/). The mutants and their progenies were genotyped by PCR using gene-specific primers and T-DNA-specific primers. (Additional File 1: Table S9) Seeds were sterilized and placed on half-strength Murashige and Skoog (½MS) medium. After cold stratification of seeds in the dark at 4 °C for 2 days, plates were transferred to the growth chamber. Plants were grown under a long day (16 h light/8 h dark) photoperiod at 20–22 °C.

### Preservation and use of strains

The *Pst* DC3000 strain was derived from long-term preservation in the laboratory and activated on a PSA (potato-sugar-agar) medium. The *Pst* DC3000(avrRpt2) strain was activated on a PSA medium with tetracycline (100 mg/mL). The D36E strain was activated on a PSA medium with spectinomycin (100 mg/mL). All strains were stored at 4 °C and activated on a medium containing the corresponding antibiotic for 24 h before use.

### Generation of transgenic plants

To rescue *atsnu13* mutants with the wild-type *AtSNU13* gene, a 2913-bp fragment spanning the *AtSNU13* gene, including the predicted 2000-bp promoter sequence, and all exons and introns sequences were amplified by PCR from wild-type plants and cloned into Pcambia1300 destination vector to generate the pro*AtSNU13*:: *AtSNU13*genomic–mcherry construct. To generate the proRBOHD::GFP-RBOHD^CDS^, proALD1::GFP- ALD1^CDS^constructs, we first cloned their promoter region (before the start codon of genes) into vector pCX-GFP-P using the restriction sites Xcm1 and add a “*CCCGGG*” in the bottom of the reverse primers as restriction sites Sma1. Then, the full-length CDSs of target genes were cloned into the vectors that carried the promoter of their promoters. These constructs were stably transformed into *atsnu13* plants using the floral dip method, respectively. Transgenic plants were selected on hygromycin and validated by PCR.

### Yeast two-hybrid assay and pull-down assay

The full-length CDSs of *AtSNU13* and *AtU4/U6.U5* were cloned into the pGADT7 AD and pGBKT7 vectors, respectively. Then, the two constructs were co-transformed into Y2HGold yeast cells (Transgen Biotech). Expression of pGADT7-T + pGBKT7-53 and pGADT7-T + pGBKT7-lam were used as a positive control. The empty vector pGADT7 AD was co-transformed as a negative control. The interaction was determined on synthetically defined (SD)/ − Ade/ − His/ − Leu/ − Trp medium following the manufacturer’s protocols (Clontech).

Purified MBP-AtSNU13 and MBP proteins were mot immunoprecipitation with MBP beads. Mot immunoprecipitation proteins were incubated with his-AtU4/U6.U5 tri-snRNP 27 K, and anti-his antibody was used to detect his-AtU4/U6.U5 tri-snRNP 27 K.

### Subcellular localization

To determine the subcellular localization of AtSNU13 and AtU4/U6.U5 tri-snRNP 27 K, the full-length CDS was cloned into the pCAMBIA1300 and transformed into *A. tumefaciens* strain GV3101, and the bacterial suspension (OD600 = 0.5) was infiltrated into *N. benthamiana* leaves with Agrobacterium carrying p19. Fluorescence was examined under a two-photon laser confocal microscope (Zeiss LSM880 NLO) 3 days after transformation.

### Bimolecular fluorescence complementation (BiFC) assays

The coding regions of *AtSNU13* and *AtU4/U6.U5 tri-snRNP 27 K* were cloned into the pSPYCE-35S and pSPYNE-35S vectors, respectively. Each was fused separately to the C-terminal half and the N-terminal half of yellow fluorescent protein (YFP); YFP^c^ and YFP^n^ were used alone as controls. These constructs were transformed into *A. tumefaciens* strain GV3101 and co-infiltrated into *N. benthamiana* leaves. Fluorescence was examined under a two-photon laser confocal microscope (Zeiss LSM880 NLO) 3 days after transformation.

### RNA extraction and RT–qPCR analysis of gene expression

Total RNA was extracted from Arabidopsis leaves using TRIzol reagent (Invitrogen, USA). Total RNA (2 μg) was reverse transcribed into cDNA using the Fast-King gDNA Dispelling RT SuperMix kit (TIANGEN). For all genes considered in this paper, ~ 5 ng of cDNA template was used for quantitative real-time PCR (qPCR). The expression of genes was normalized by the eq. 2-ΔCT, where ΔCT is the CT target gene–CT ACTIN. The CFX96 Real-Time PCR detection system (Bio-Rad) was used for all qPCRs with three technical repeats.

### ROS detection

Whole seedlings were placed into a 1% (M/V) sodium azide solution, vacuum-infiltrated for 30 min, then transferred into a 0.5 mg/ml nitro blue tetrazolium (NBT) solution, followed by another vacuum-infiltration for 30 min. Sodium azide may improve cell permeability, allowing the NBT solution to spread throughout the entire seedling. NBT reacted with O^2−^ forming a dark blue insoluble complex substance. The excess dye solution was washed away with boiled ethanol, and the whole seedlings were imaged by an SMZ25 stereoscopic microscope (Nikon, Japan). The accumulation of hydrogen peroxide (H_2_O_2_) in leaves was visualized histochemically using 3,3-diaminobenzidine (DAB) as an indicator. The seedlings were put into 1 mg/ml DAB solution, vacuum-infiltrated for 30 min, washed three times with deionized water, and reacted with H_2_O_2_ for 8 h under light at 28 °C. After the reaction, a deep brown deposition was visible in leaves, and excess dye solution was washed away with boiled ethanol and seedlings were imaged at length using a stereomicroscope.

The sterilized water (mock), 100 nM flg22, 5 μM dexamethasone, or 100 nM flg 22 + 5 μM dexamethasone was added to the leaf discs, and ROS burst was recorded. For detection of ROS production by 2′,7′-dichlorofluorescein diacetate (DCF-DA) (Solarbio, CA1410) under confocal microscopy, plants were infiltrated with D36E (OD_600_ = 0.02) or D36E(avr*Rpt2*) (OD_600_ = 0.02), air-dried and placed into the plant growth room. DCF-DA can pass through the cell membrane freely, and after entering the cell, it can be hydrolyzed by the intracellular esterase to produce DCFH. Intracellular reactive oxygen species can oxidize non-fluorescent DCFH to produce fluorescent DCF. Detecting the fluorescence of DCF can determine the level of intracellular ROS. Ten micromolar DCF-DA solution was infiltrated into the leaf and fluorescence signal was detected 10 min later. Fluorescence was examined under a two-photon laser confocal microscope (Zeiss LSM880 NLO), using a 488-nm excitation wavelength and 525 nm emission wavelength.

### Callose accumulation detection

The 22-day *Arabidopsis thaliana* were treated with flg22 for 24 h; the leaves were placed in a solution containing 10% lactic acid, 10% phenol, 4% glycerin, 50% ethanol (v/v) and vacuumed for 30 min and then decolorized to colorless. Transfer to 150-mM dimethyl bisulfate solution containing 1/1000 aniline blue powder overnight was performed. Callose deposition was imaged using a Zeiss LSM880 laser scanning microscope. The excitation wavelength was 405 nm, and the emission wavelength was 415–485 nm. Fluorescence intensity was quantified using the ImageJ software.

### Microbe cultivation assays

All the Arabidopsis seedlings used for the incubation of *Pseudomonas syringae pv.* tomato (*Pst*) DC3000 were grown for 4 weeks. *Pst* DC3000 was grown on PSA medium containing 50 μg/ml rifampicin at 28 °C. *Pst* DC3000 was eluted from the PSA culture medium using 10 mM MgCl_2_. The optical density of the bacterial suspension reached OD_600_ at an absorbance wavelength of ~ 0.002. The bacterial suspension was infiltrated into 4-week-old Arabidopsis using a needleless syringe. Bacterial growth assay was performed at 0 and 3 days after incubation of the bacterial suspension. The diseased leaves were surface sterilized in 75% ethanol solution for 60 s and then washed 3 times using sterile distilled water. A mortar was used to grind leaves into homogenate in sterile distilled water, continuous dilution (1:10) of the homogenate was performed, and the homogenate was plated on PSA medium for calculating single colonies after growing at 28 °C for 1–2 days.

### RNA-seq analysis

RNA samples were taken from 14-day-old Arabidopsis leaves in wild-type and *atsnu13* mutant plants, which were injected with *Pst* DC3000, *Pst* DC3000 (Rpt2), and D36E. RNA samples were selected depending on quality (RIN score ≥ 7). Samples were pooled and sequenced by Wuhan Genomic Institution (www. genomics.org.cn, BGI, Shenzhen, China). Clean tags were mapped to the reference genome in the Arabidopsis TAIR10.2 reference genome, and genes were available. The quality statistics of filtered reads, filtering components of the original data, and comparison of reference genomes were analyzed (Additional File 1: Tables S2-S4). The original sequence data has been uploaded to the NCBI Sequence Read Archive (https://trace.ncbi.nlm.nih.gov/Traces/sra/). All differential gene expressions were based on the following standard: the absolute value of log_2_ ratio ≥ 1 and FDR ≤ 0.001.

### Validation of AS events and measurement of splicing ratio

In brief, the splicing ratio was calculated by determining the level of spliced RNA normalized to the level of unspliced RNA for each intron tested using the eq. 2Δ (Ct-unspliced-Ct-spliced). The splicing ratio of the wild-type strain was normalized to 1. For amplifying the spliced RNA, the forward primer was designed to cross exon-exon junctions to ensure specific amplification of spliced RNA, and the reverse primer was designed to target the exon adjacent to the intron. For amplifying the unspliced RNA, the forward primer was designed within the intron, and the reverse primer was the same as that for amplifying the spliced RNA, as described previously [77]. AS is analyzed through ASpli, which combines statistical information from exon, intron, and splice junction differential usage (*P*-value, FDR) with information from splice junction reads to calculate differences in the percentage of exon inclusion (∆PSI) and intron retention (∆PIR). For ES, A3SS, and A5SS events, exon inclusion and exclusion splice forms were determined. For IR events, intron exclusion and inclusion splice forms were determined. Then, the difference in splicing ratios (*R* = FPKM_on_/(FPKM_on_ + FPKM_off_) between wild-type and mutant samples was calculated as Δ =|*R*_WT_ − *R*_mutant_| for each event [78].

### Electrophoretic mobility shift assay

Full-length CDSs of *AtSNU13* and *AtU4/U6.U5* were amplified and cloned into pMal-c2X and pET-28a. The fusion proteins were expressed in *E. coli* BL21 (DE3) and purified to homogeneity using a Ni–NTA (Roche, USA) resin column. The oligonucleotides containing elements were synthesized by Sangon Biotech (China) with 5′-biotin marked. Briefly, biotin-labeled probes were incubated with single AtU4/U6.U5 and AtSNU13 and AtU4/U6.U5 complex at room temperature for 20 min, and the subsequent steps were performed using a Chemiluminescent EMSA Kit (Beyotime, China) according to the manufacturer’s instructions. Free and bound probes were separated via PAGE, transferred to nylon membranes, and subjected to chemiluminescence examination.

### Supplementary Information

Additional file 1: Table S1. Sequence of AtSNU13 homologs. Table S2. The quality statistics of filtered reads. Table S3. The quality statistics of filtering components of the original data. Table S4. The quality statistics of comparison of reference genomes. Table S5. Different expression genes in *atsnu13* were detected by RNA-Seq analysis. Table S6. Different expression genes in plant defense in atsnu13 were detected by RNA-seq analysis. Table S7. Statistics of AS events detected in all lines. Table S8. Summary of differential splicing events for all comparison groups. Table S9. Primers used in this study. Table S10. The original data used in this study.

Additional file 2: Fig. S1 T-DNA insertion sites and expression of *AtSNU13* transcripts in *atsnu13* plants. Fig. S2 Appearance of the 5-week-old *atsnu13* mutant plants before bacteria inoculation. Fig. S3 Phylogenetic analysis and schematic representation of the domain architecture of homologs of the SNU13 protein. Fig. S4 Preparation of transgenic and the expression of transcripts in *Arabidopsis thaliana.* Fig. S5 The expression pattern of *AtSNU13.* Fig. S6 The difference of between H_2_O_2_ and O^2−^ accumulate in the wild-type and *atsnu13.* Fig. S7 Quantification of callose deposition in leaves. Fig. S8 Analysis of RNA-seq in Col-0 and *atsnu13.* Fig. S9 The transcription expression of several defense-related genes found in RNA-seq. Fig. S10 Independent validation of splicing events detected by RNA-seq. Fig. S11 The qualities of the protein preparations used in EMSA assay. Fig. S12 DAB and NBT staining in transgene lines. Fig. S13 The rRNA in the Col-0, *atsnu13,* and *atsnu13*/ + *AtSNU13* lines. Fig. S14 Detection of atsnu13/RBOHD and atsnu13/ALD1 transgenic lines. Fig. S15 The original image of Western Blotting used in the article.

## Data Availability

All data supporting the findings of this study are available within the article and within its supplementary materials published online. Illumina Sequencing data have been submitted to the National Center for Biotechnology Information (NCBI) Sequence Read Archive (SRA) under Bio-Project: PRJNA909008.

## Abbreviations

PAMPs or MAMPs: Pathogen-associated molecular patterns
PTI: PAMP-triggered immunity
PRRs: Pattern recognition receptors
R: Resistance
AVR: Avirulence
NBS: Nucleotide-binding site
ETI: Effector-triggered immunity
NMD: Nonsense-mediated mRNA decay
snRNPs: Small nuclear ribonucleoproteins
SR: Serine/arginine-rich
hnRNPs: Heterogeneous nuclear ribonucleoproteins
RBPs: RNA-binding proteins
IME: Intron-mediated enhancement
avrRpt2: Strains carrying RPT2 effectors in *Pst* DC3000
D36E: All 36 effector genes and coronatine biosynthesis genes are deleted in *Pst* DC3000
PCR: Polymerase chain reaction
Y2H: Yeast two-hybrid
BIFC: Bimolecular fluorescence complementation
DAB: 3,3-Diaminobenzidine
NBT: Tetranitro blue tetrazolium chloride
ROS: Reactive oxygen species
PSI&PIR: Percentage of exon inclusion and intron retention
EMSA: Electrophoretic mobility shift assay
