# Supplementary material for: AtSNU13 modulates pre-mRNA splicing of RBOHD and ALD1 to regulate plant immunity
Source: BMC Biol. 2024 Jul 10;22:153. doi: 10.1186/s12915-024-01951-9 (PMC11234627; doi:10.1186/s12915-024-01951-9)
Supplement: Supplementary file 2 — Additional file 2: Fig. S1 T-DNA insertion sites and expression of AtSNU13 transcripts in atsnu13 plants. Fig. S2 Appearance of the 5-week-old atsnu13 mutant plants before bacteria inoculation. Fig. S3 Phylogenetic analysis and schematic representation of the domain architecture of homologs of the SNU13 protein. Fig. S4 Preparation of transgenic and the expression of transcripts in Arabidopsis thaliana. Fig. S5 The expression pattern of AtSNU13. Fig. S6 The difference of between H2O2 and O2− accumulate in the wild-type and atsnu13. Fig. S7 Quantification of callose deposition in leaves. Fig. S8 Analysis of RNA-seq in Col-0 and atsnu13. Fig. S9 The transcription expression of several defense-related genes found in RNA-seq. Fig. S10 Independent validation of splicing events detected by RNA-seq. Fig. S11 The qualities of the protein preparations used in EMSA assay. Fig. S12 DAB and NBT staining in transgene lines. Fig. S13 The rRNA in the Col-0, atsnu13, and atsnu13/ + AtSNU13 lines. Fig. S14 Detection of atsnu13/RBOHD and atsnu13/ALD1 transgenic lines. Fig. S15 The original image of Western Blotting used in the article. [file 12915_2024_1951_MOESM2_ESM.docx]

**
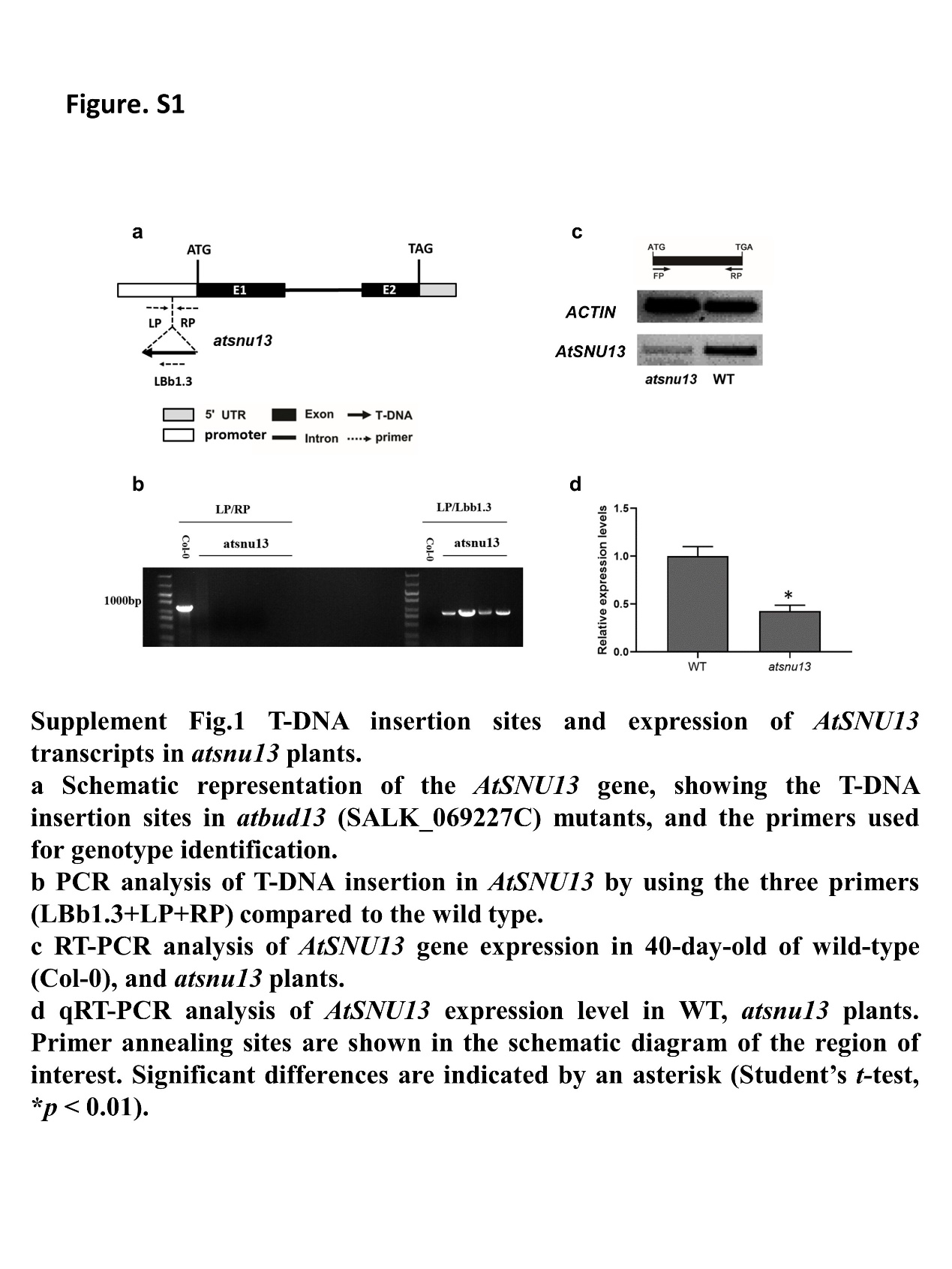
**

**Fig. S1** **T-DNA insertion sites and the expression of AtSNU13 transcripts in *atsnu13* plants.**

a schematic representation of the *AtSNU13* gene, showing the T-DNA insertion sites in *atsnu13* (SALK_069227C) mutants and the primers used for genotype identification.

b PCR analysis of *atsnu13* mutant plants bearing a T-DNA insertion by using three primers (LBb1.3+LP+RP) compared to wild-type plants.

c RT-PCR analysis of the expression of the *AtSNU13* gene in 40-day-old wild-type (Col-0) and *atsnu13* plants.

d RT-qPCR analysis to quantify the expression level of the *AtSNU13* gene in WT and *atsnu13* plants; The annealing sites of primers are shown in the schematic diagram of the region of interest. Significant differences are indicated with an asterisk (* p < 0.01; Student’s t-test).

 
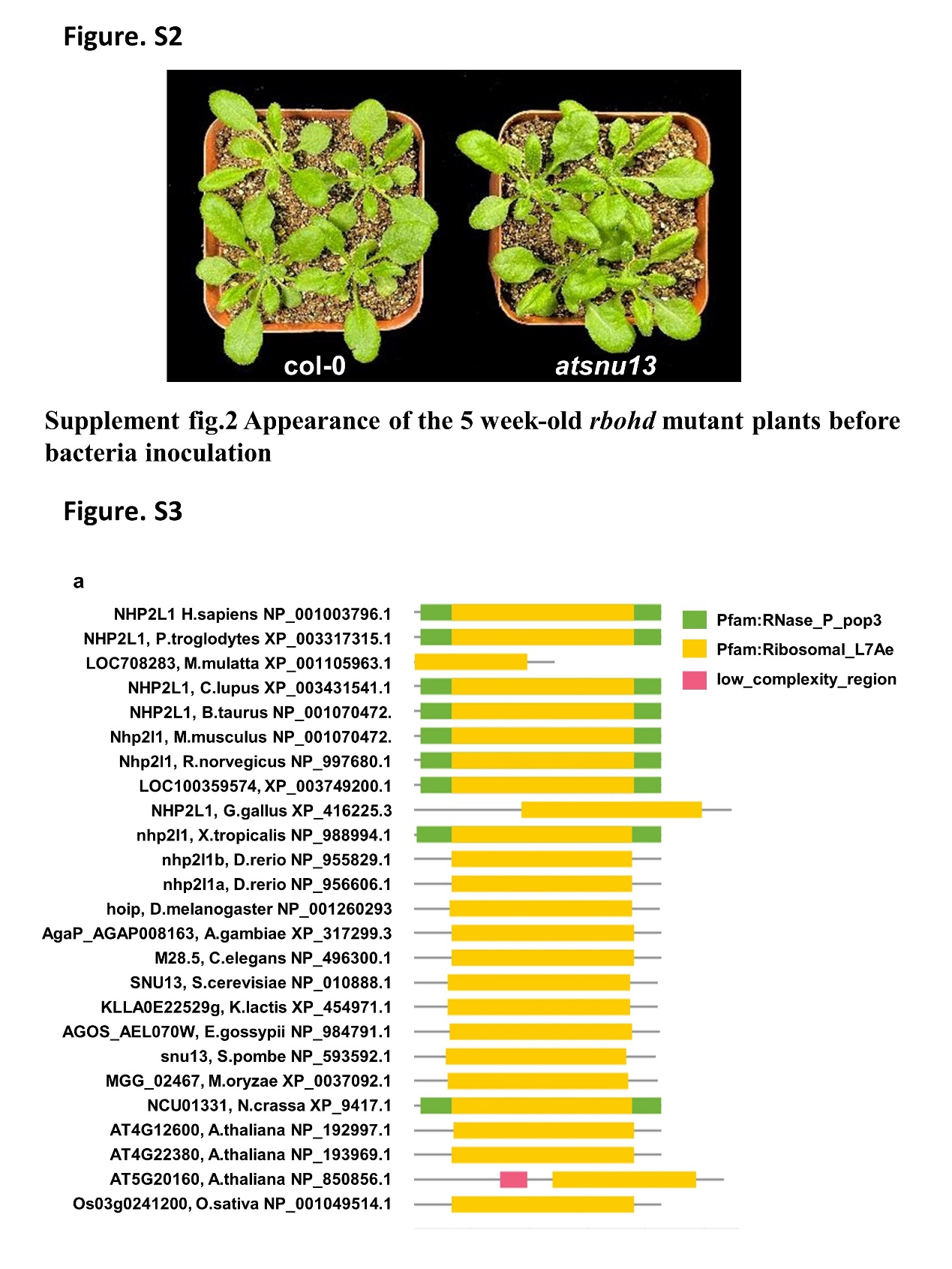


**Fig. S2 The appearance of the 5-week-old *atsnu13* mutant plants before inoculation with pathogenic bacteria.**

 
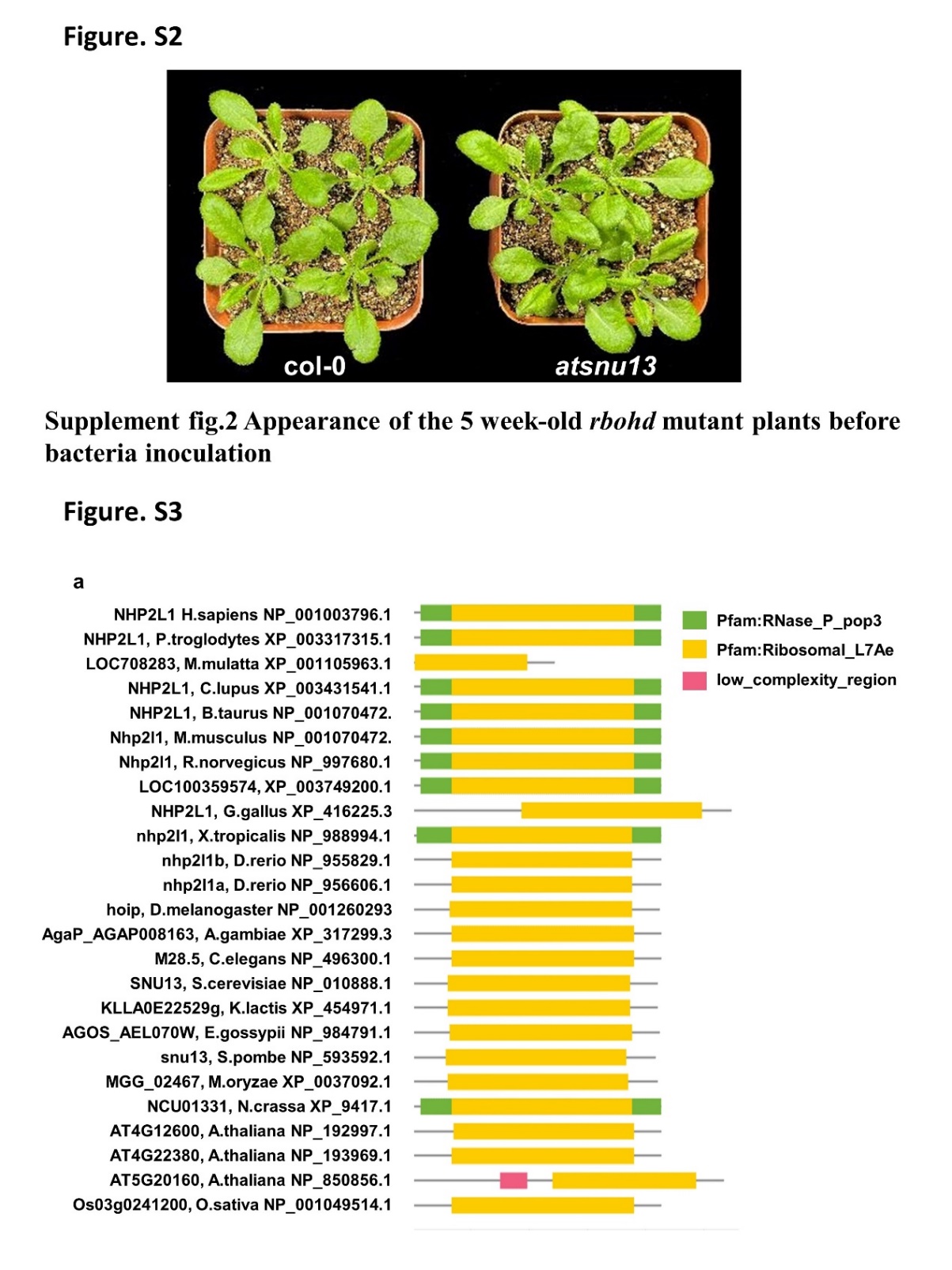


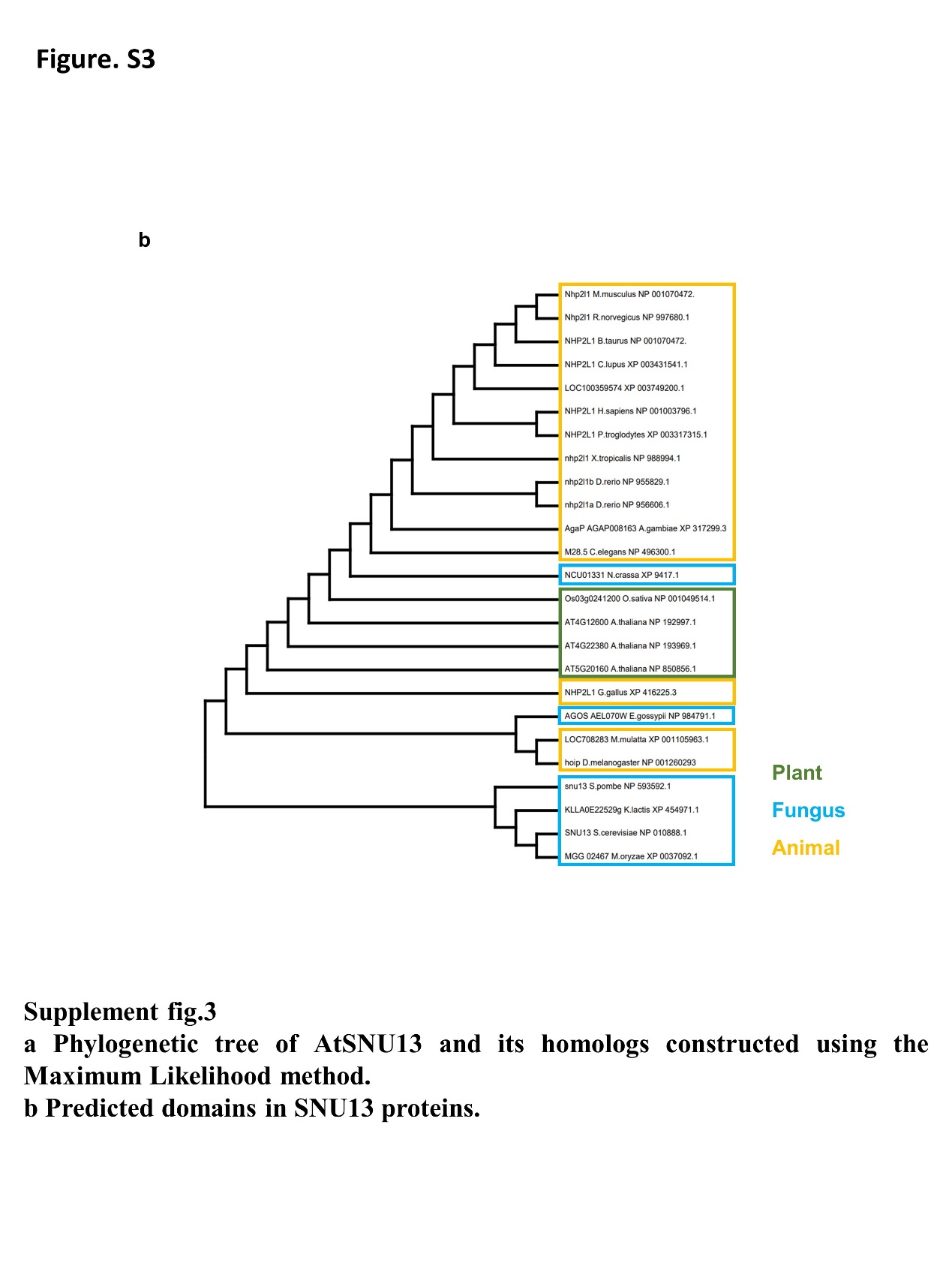


**Fig. S3 Phylogenetic analysis and schematic representation of the domain architecture of homologs of the SNU13 protein**

a The phylogenetic tree of AtSNU13 and its homologs, constructed using the maximum likelihood method

b Predicted domains in homologs of the SNU13 protein


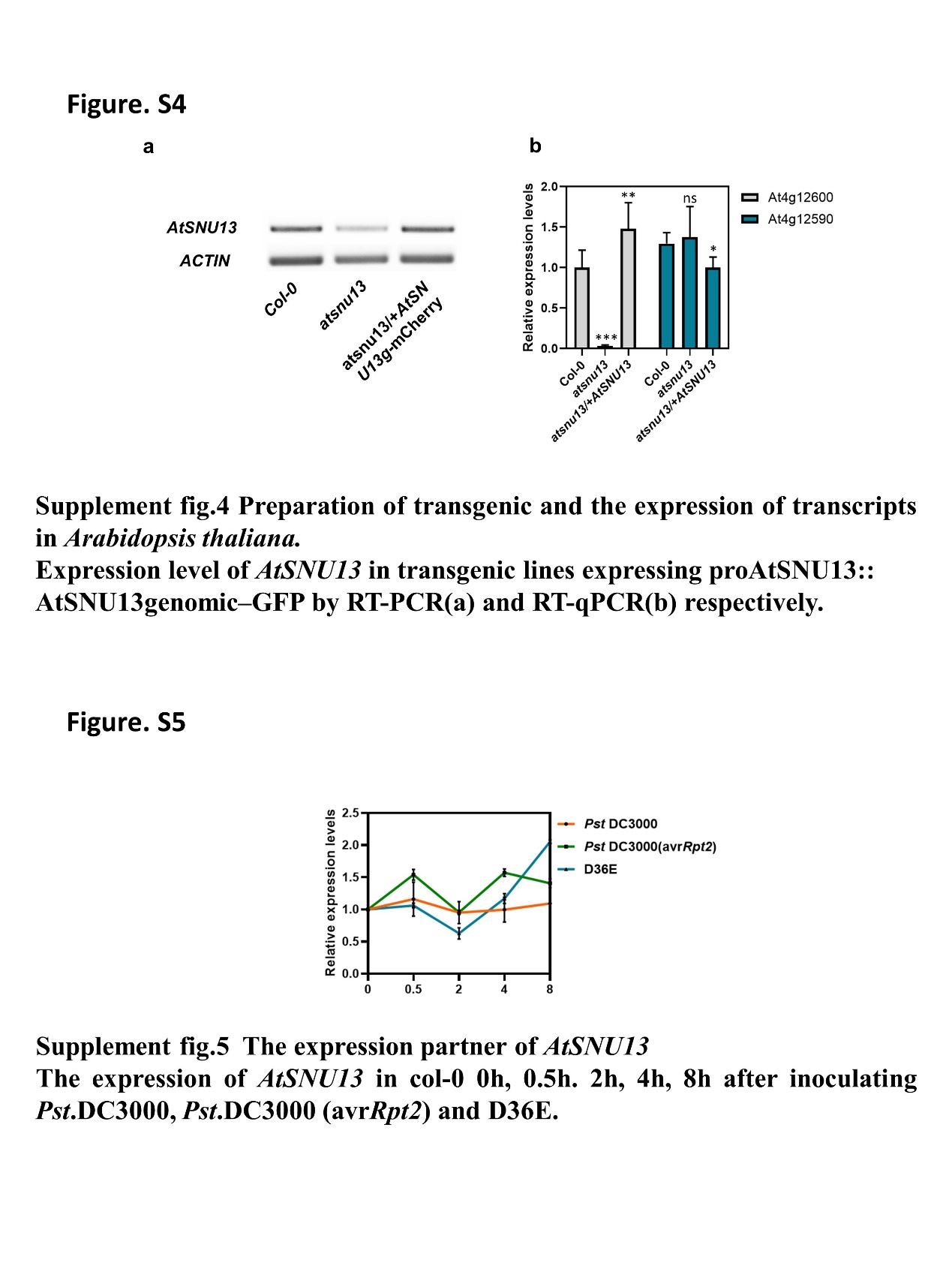


**Fig. S4 T-DNA insertion sites and the expression of *AtSNU13* transcripts in *atsnu13* and *atsnu13*/+*.***

Expression level of *AtSNU13* and At4g12590 in transgenic lines expressing pro*AtSNU13*:: *AtSNU13*genomic–GFP by RT-PCR(a) and RT-qPCR(b) respectively**.**


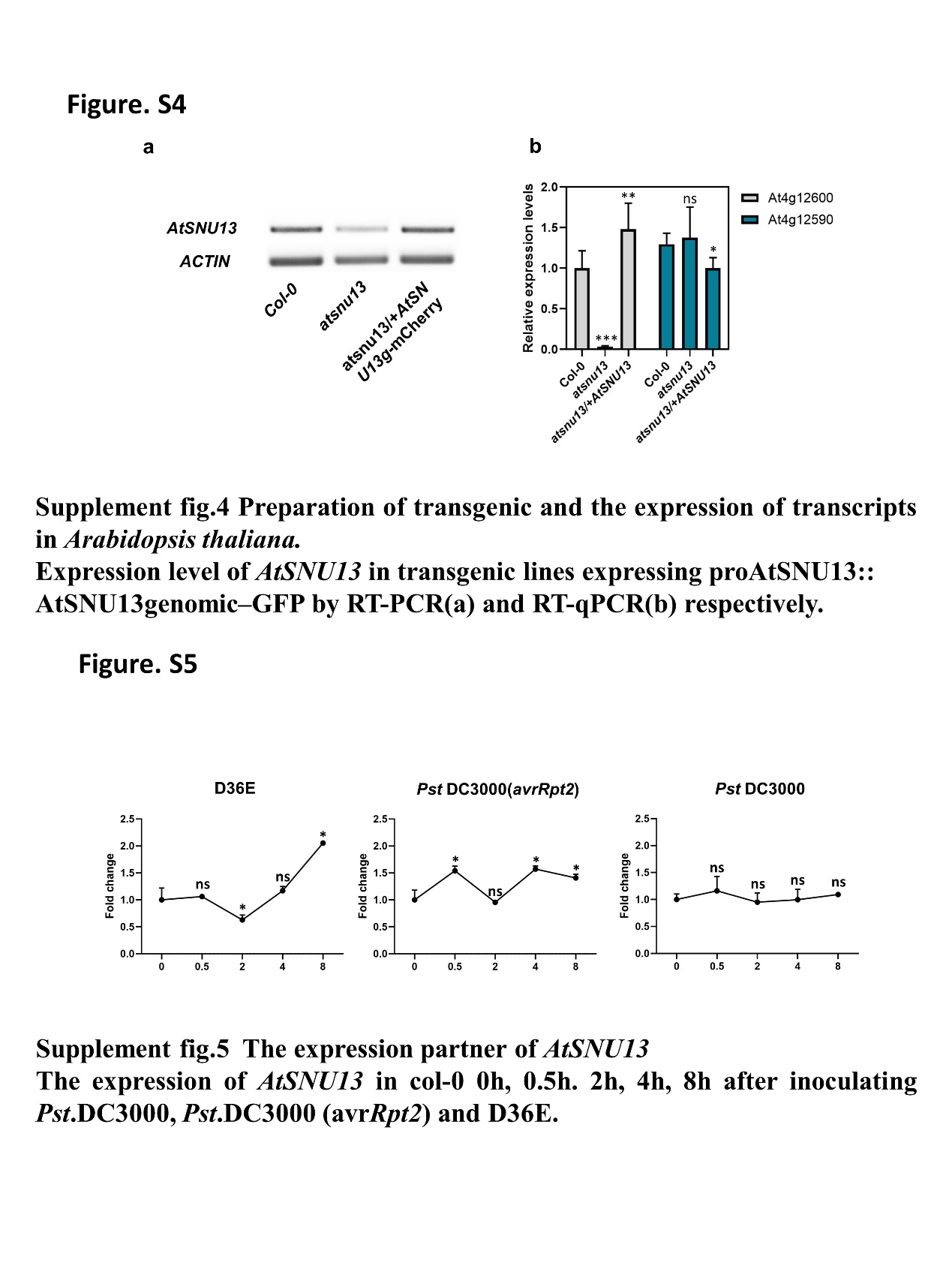


**Fig.S5 The expression pattern of *AtSNU13***

The expression of *AtSNU13* in Col-0 0 h, 0.5 h, 2 h, 4 h, 8 h after inoculating *Pst* DC3000, *Pst* DC3000 (avr*Rpt2*) and D36E.


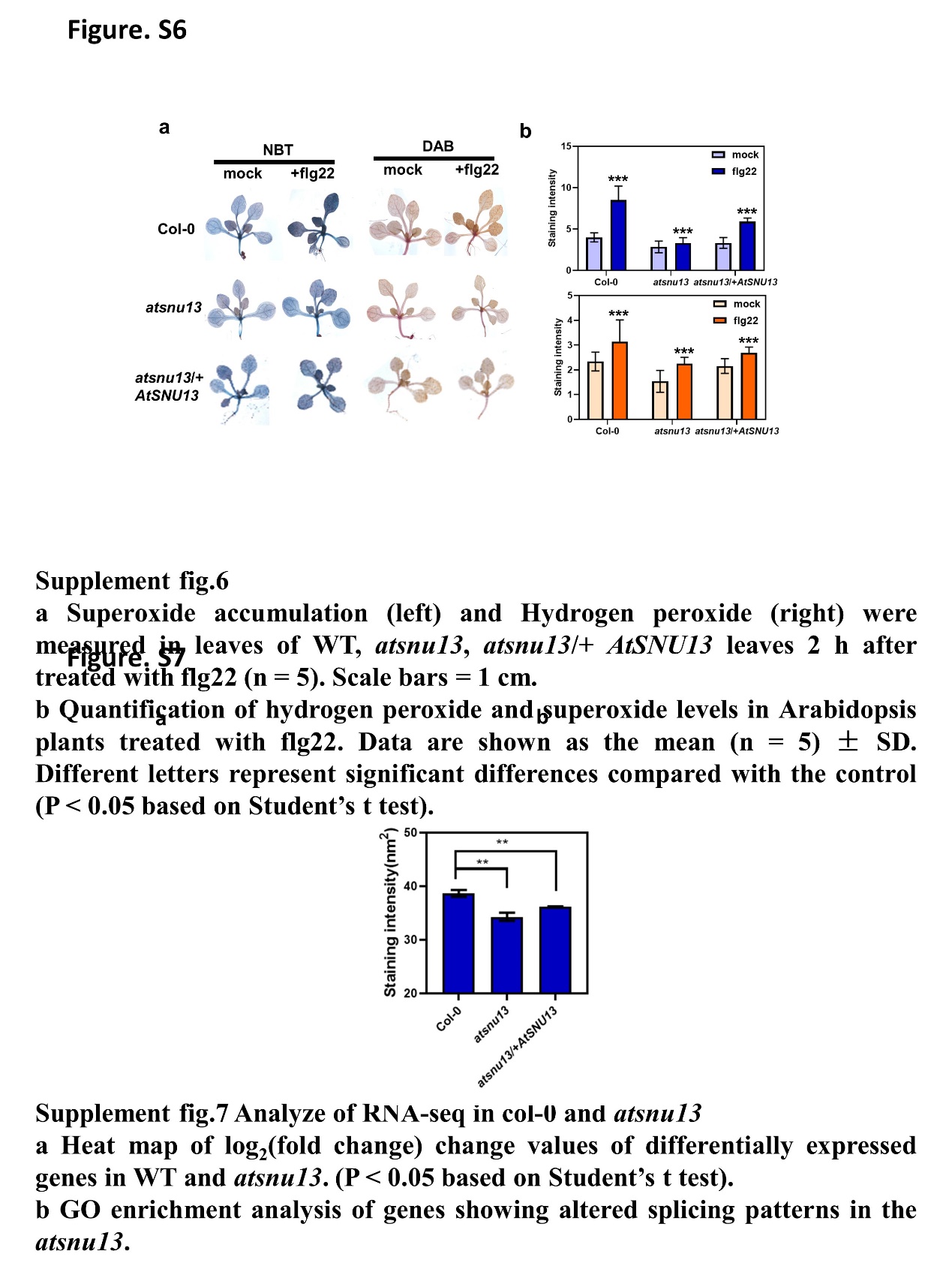


**Fig.S6** **The difference between H_2_O_2_ and O^2-^ accumulate in the wild-type and *atsnu13***

a Superoxide accumulation (left) and Hydrogen peroxide (right) were measured in leaves of WT, *atsnu13*, *atsnu13*/+ *AtSNU13* leaves 2 h after treated with flg22.

b Quantification of hydrogen peroxide and superoxide levels in Arabidopsis plants treated with flg22. Data are shown as the means (n = 3) ± S.E.M. Different letters represent significant differences compared with the control (P < 0.05 based on Student’s t test).


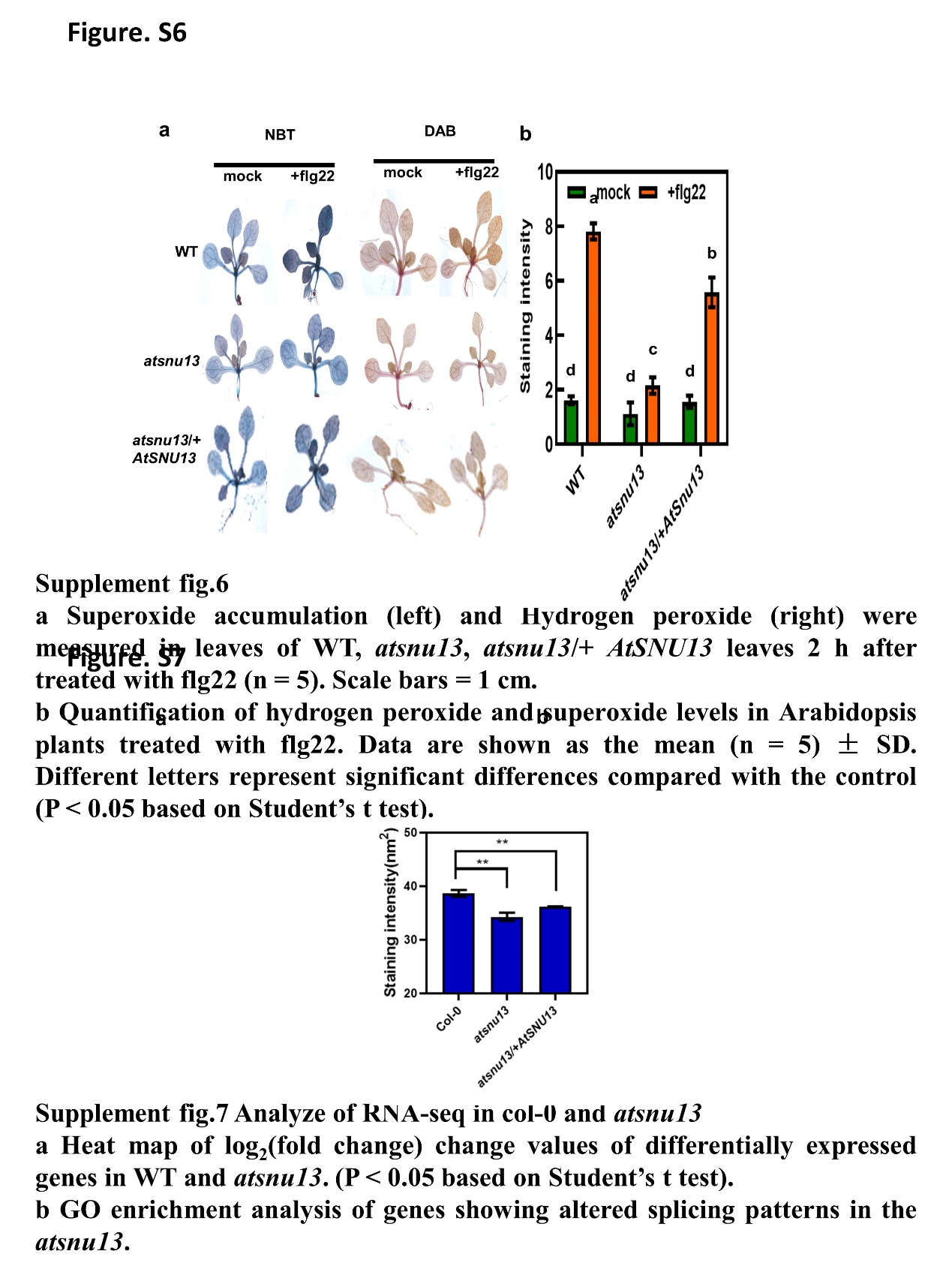


**Fig.S7 Quantification of** **callose deposition in leaves.**

Data represent means ±S.E.M (n = 3). Different letters indicate significant differences among treatments and the control (P < 0.05 based on Student’s t-test).


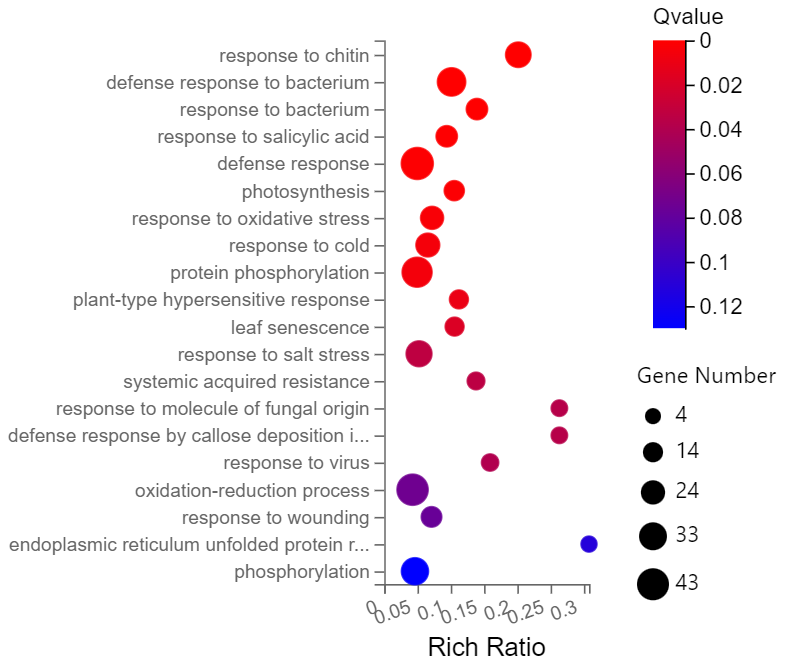


**Fig.S8 Analysis of RNA-seq in Col-0 and *atsnu13***

GO enrichment analysis of genes showing differentially expressed genes between Col-0 and *atsnu13* (P<0.05).


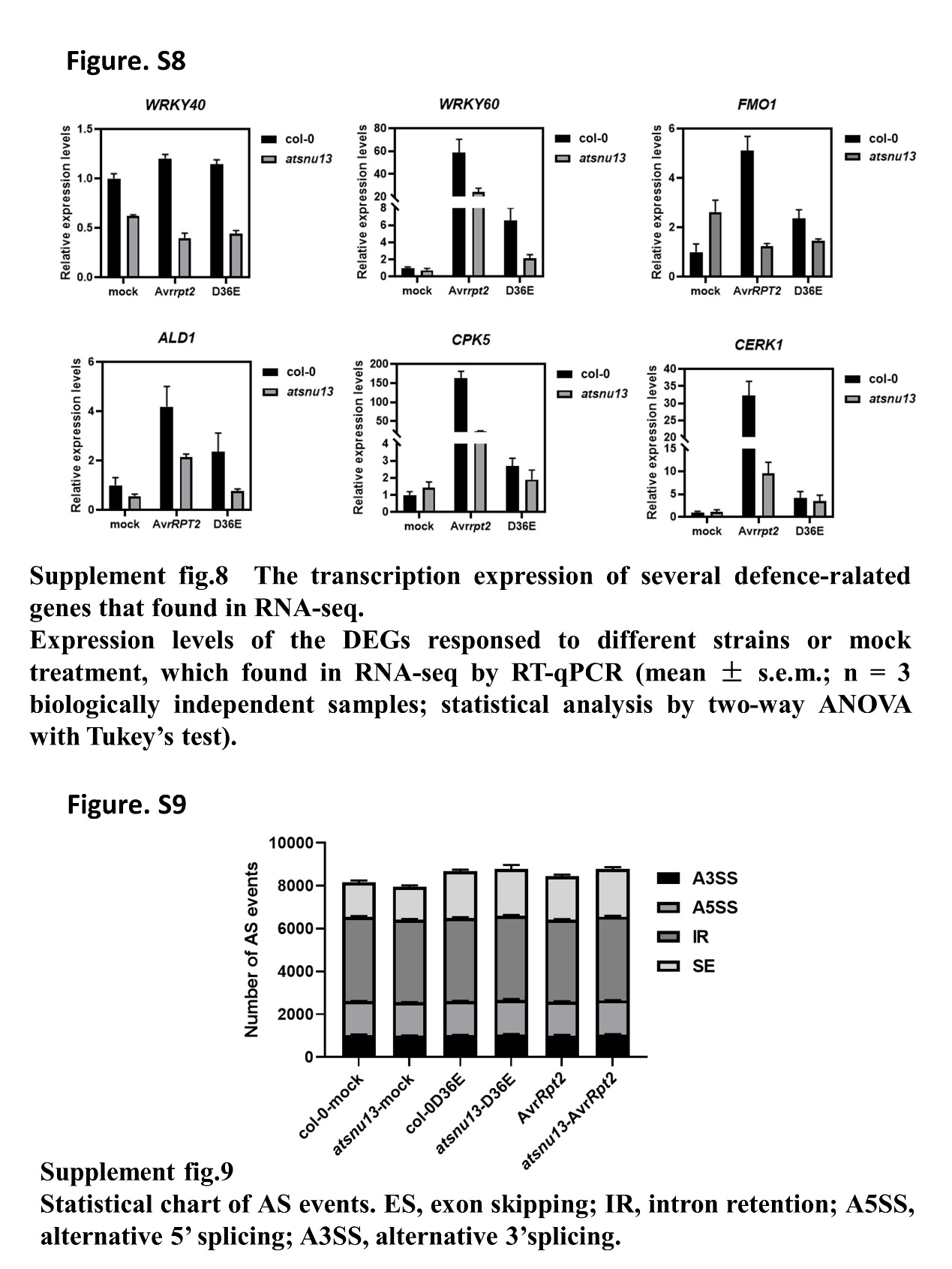


**Fig.S9 The transcription expression of several defense-related genes that found in RNA-seq.**

Expression levels of the DEGs in response to different strains or mock treatment, which found in RNA-seq by RT-qPCR (means ± S.E.M.; n = 3).

*
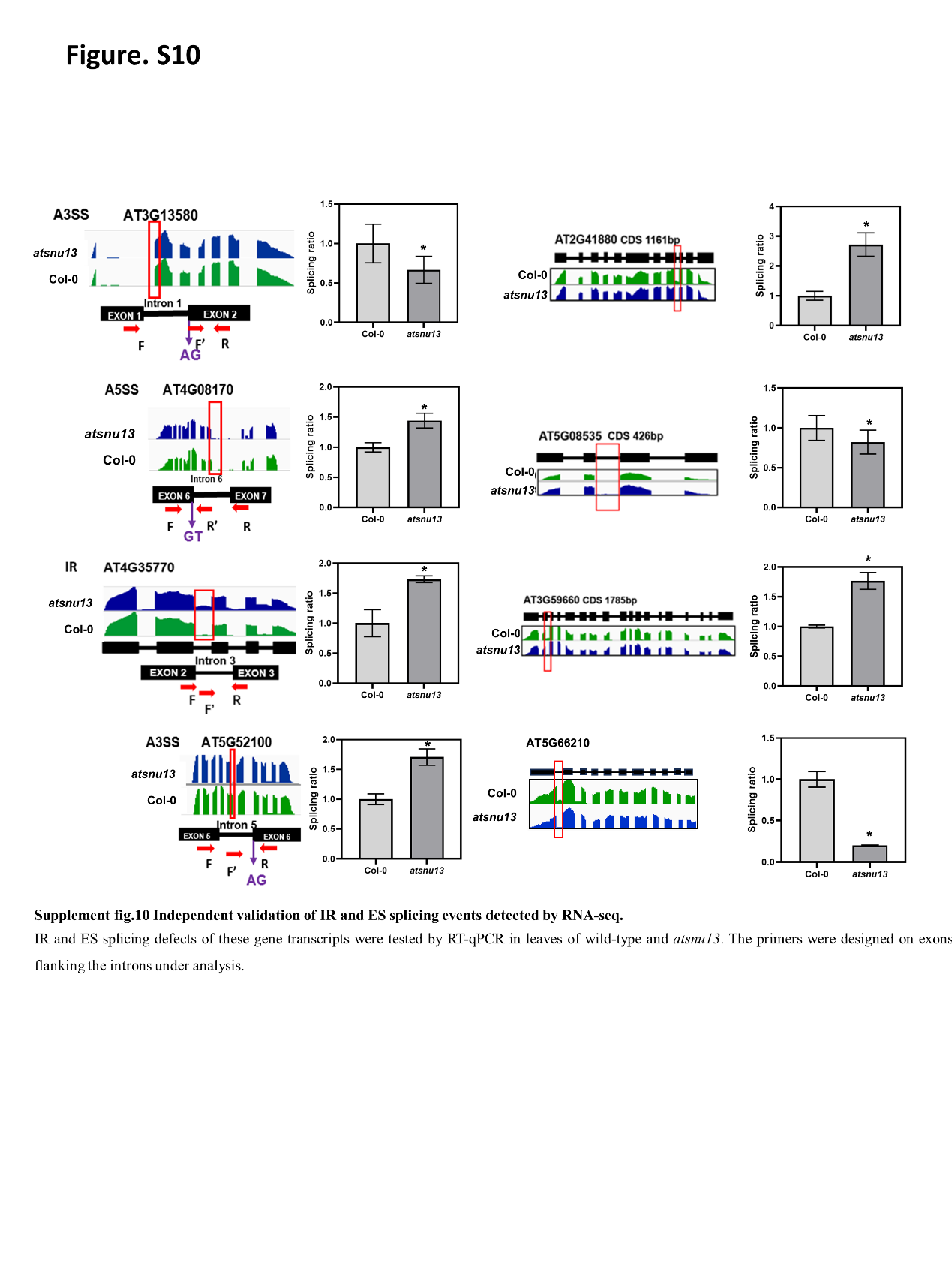
*

**Fig.S10** **Independent validation of IR and ES splicing events detected by RNA-seq.**

IR and ES splicing defects of these gene transcripts were tested by RT-qPCR in leaves of wild-type and *atsnu13*. The primers were designed on exons flanking the introns under analysis.


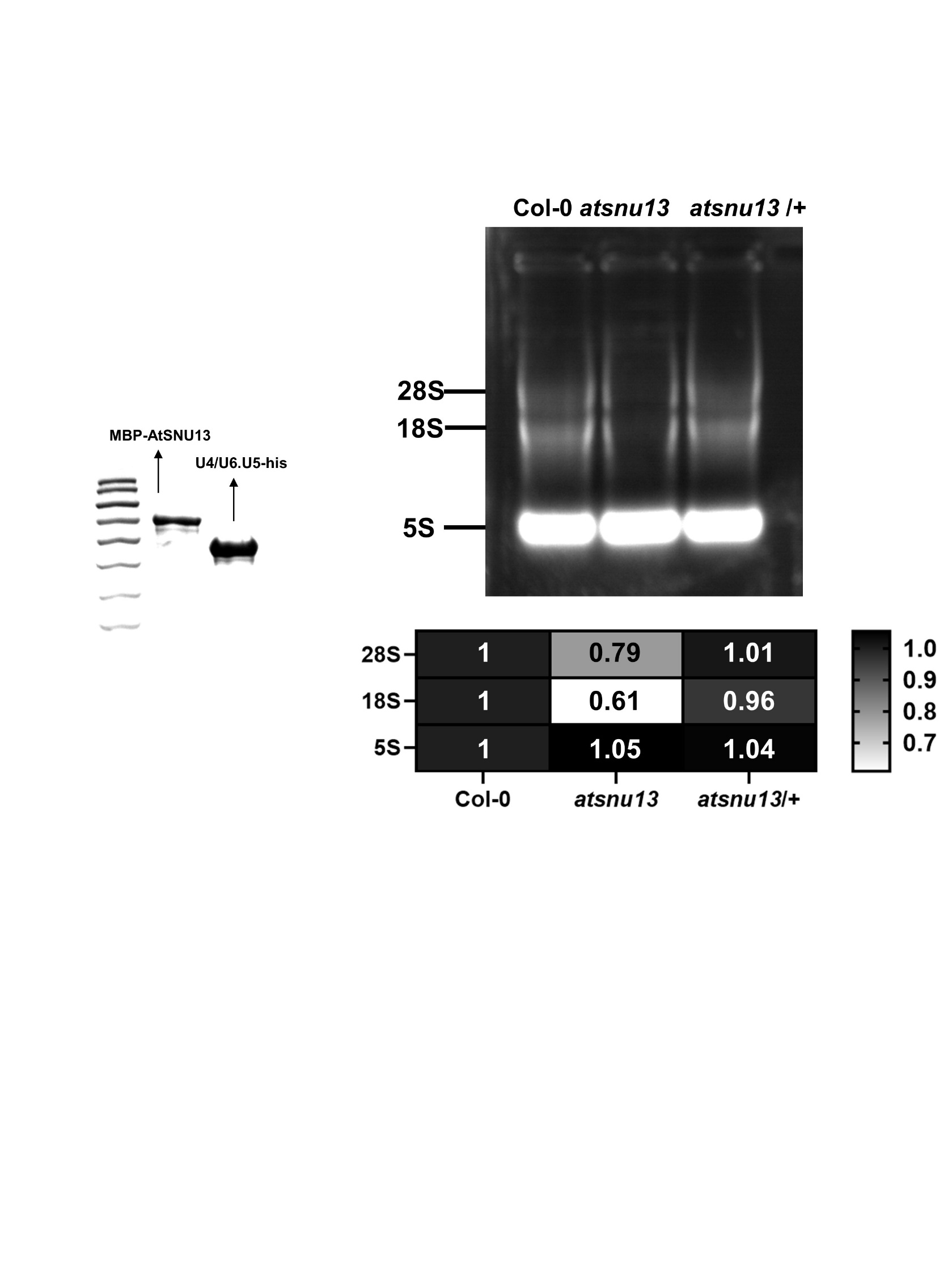


**Fig.S11 The qualities of the protein preparations used in EMSA assay.**

The MBP-AtSNU13 and U4/U6.U5-his purified by resin and used in EMSA assay.

**
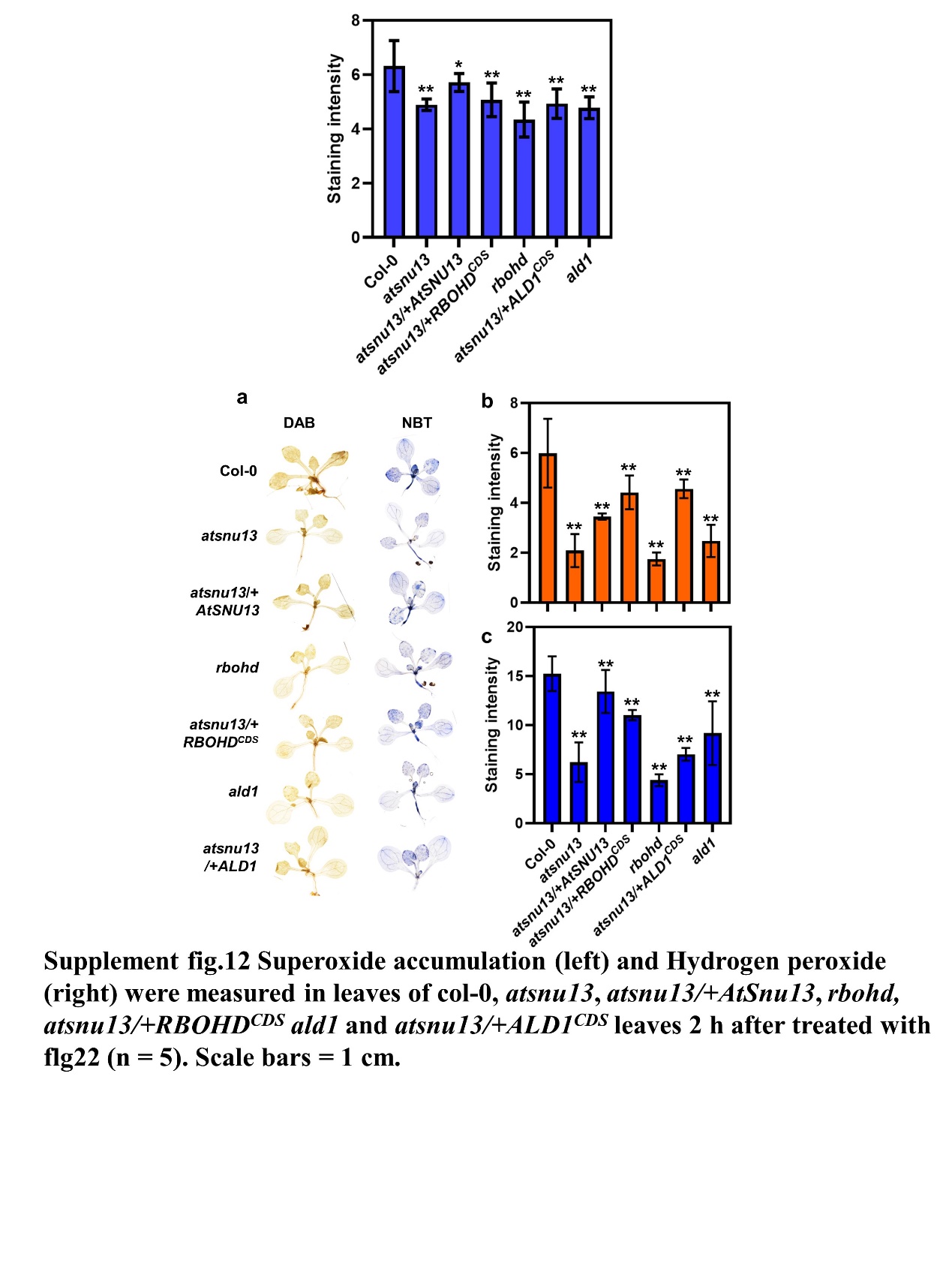
**

**Fig.S12** **DAB and NBT staining in transgene lines.**

Superoxide accumulation (left) and Hydrogen peroxide (right) were measured in leaves of Col-0, *atsnu13*, *atsnu13/+AtSnu13*, *rbohd, atsnu13/+RBOHD*，*ald1* and *atsnu13/+ALD1* leaves 2 h after treated with flg22.


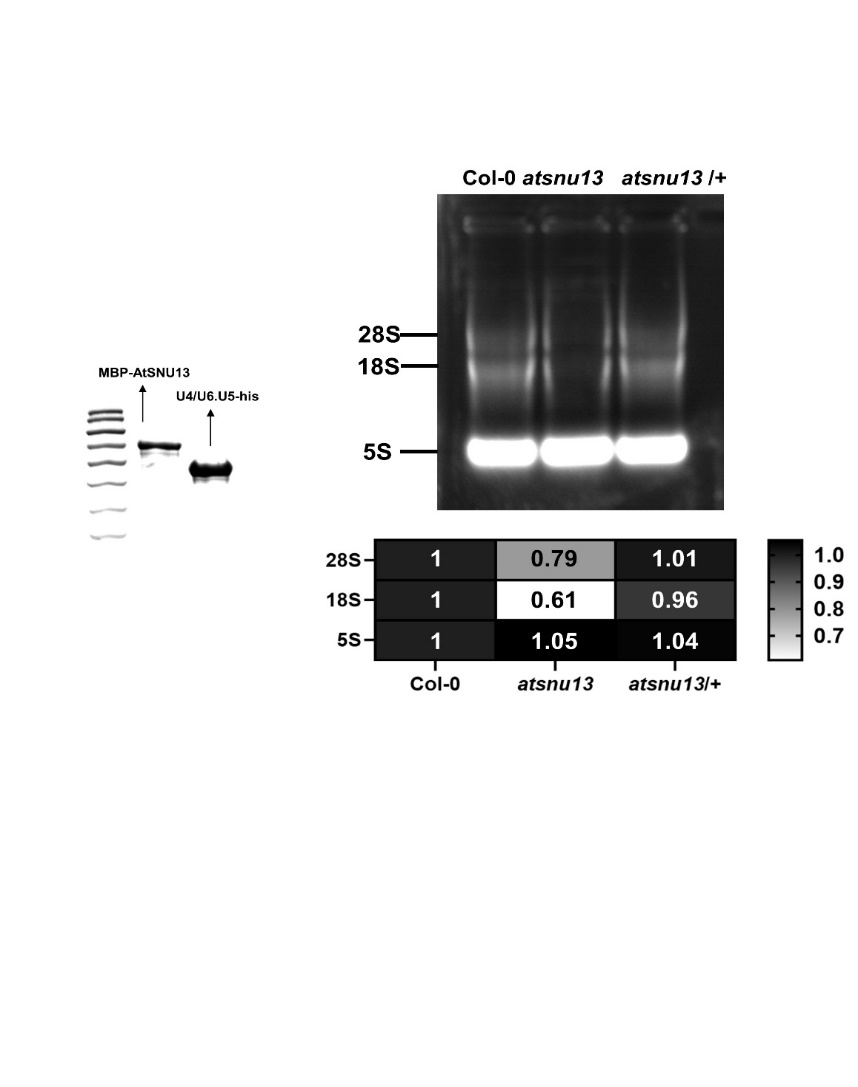


**Fig.S13 The rRNA in the Col-0, *atsnu13* and *atsnu13*/+*AtSNU13* lines.**

The total RNA was extracted and separated by gel.


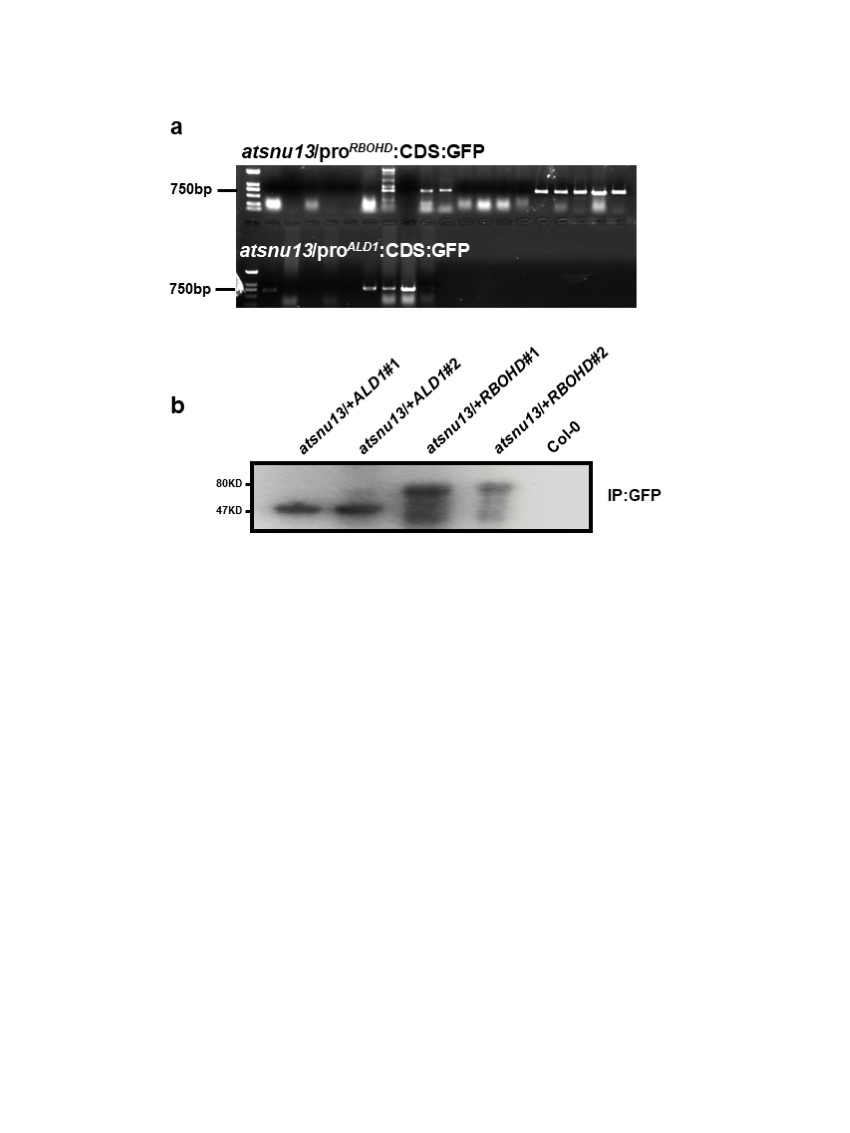


**Figure S14 Detection of *atsnu13/RBOHD* and *atsnu13/ALD1* transgenic lines.**

**a** Assessment of hygromycin resistance gene insertion in *atsnu13/RBOHD* and *atsnu13/ALD1.*

**b** Assessment of protein level expression in *atsnu13/RBOHD* and *atsnu13/ALD1.*


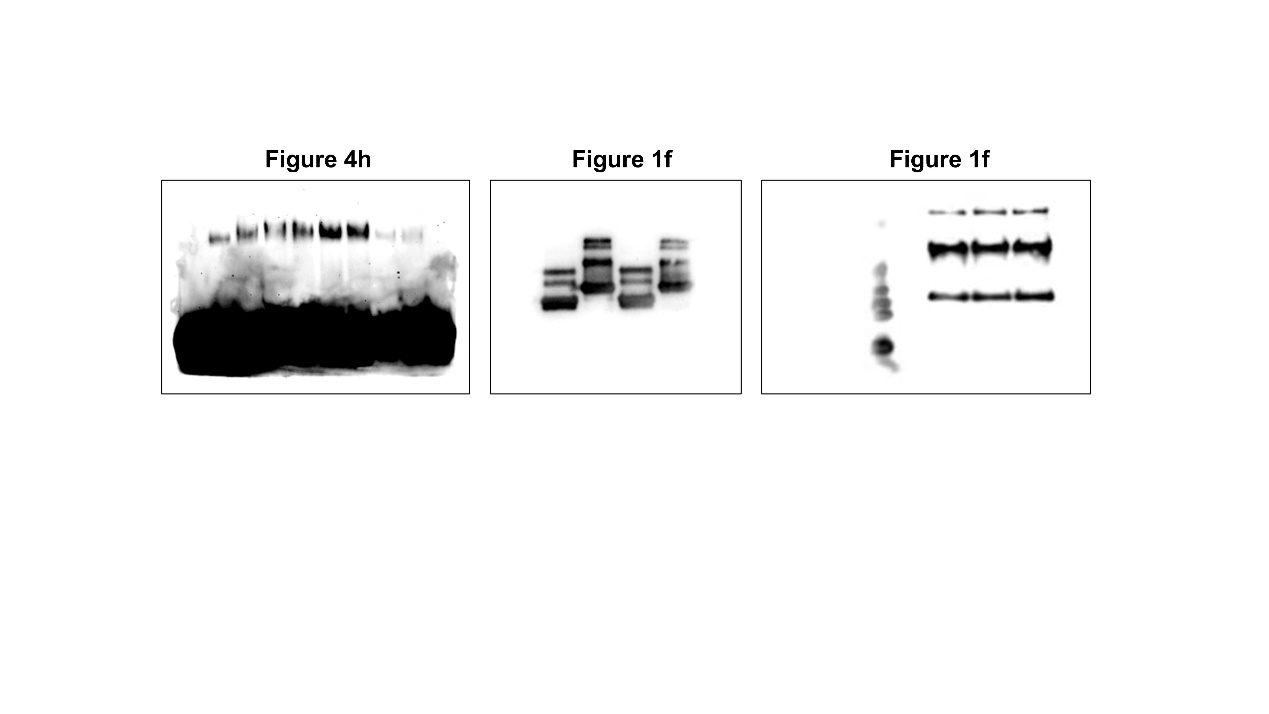


**Figure S15 The original image of Western Blotting used in the article.**
